# Serum Untargeted Metabolomics Reveal Potential Biomarkers of Progression of Diabetic Retinopathy in Asians

**DOI:** 10.3389/fmolb.2022.871291

**Published:** 2022-06-09

**Authors:** Zongyi Wang, Jiyang Tang, Enzhong Jin, Yusheng Zhong, Linqi Zhang, Xinyao Han, Jia Liu, Yong Cheng, Jing Hou, Xuan Shi, Huijun Qi, Tong Qian, Li Yuan, Xianru Hou, Hong Yin, Jianhong Liang, Mingwei Zhao, Lvzhen Huang, Jinfeng Qu

**Affiliations:** Department of Ophthalmology, Peking University People’s Hospital, Eye Diseases and Optometry Institute, Beijing Key Laboratory of Diagnosis and Therapy of Retinal and Choroid Diseases, College of Optometry, Peking University Health Science Center, Beijing, China

**Keywords:** Type 2 diabetes, proliferative diabetic retinopathy, non-proliferative diabetic retinopathy, metabolomics, untargeted metabolomics, metabolic profiling, LC-MS

## Abstract

**Purpose:** To reveal molecular mechanisms of diabetic retinopathy (DR) in Asians and facilitate the identification of new therapeutic targets through untargeted metabolomics. To determine the differences in serum metabolites and metabolic pathways between different stages of diabetic retinopathy in patients with type 2 diabetic mellitus (T2DM) and proliferative DR (PDR) and non-proliferative DR (NPDR) and identify differential metabolites between T2DM and DR (NPDR and PDR) patients.

**Methods:** This prospective observational registration study described the differential metabolites between 45 T2DM patients and 15 control cases with no significant differences in clinical characteristics. Their biospecimens and clinical information were collected and recorded in their medical reports. DR phenotypes of the subjects were verified by retina specialists. Serum metabolites were analyzed using high-resolution mass spectrometry with liquid chromatography. Untargeted metabolomics was performed on serum samples from 15 T2DM patients, 15 non-proliferative diabetic retinopathy patients, 15 proliferative diabetic retinopathy patients, and 15 diabetic controls. Discriminatory metabolic features were identified through partial least squares discriminant analysis (PLS-DA), hierarchical clustering analysis (HCA), and generalized linear regression models.

**Result:** Through untargeted metabolomics, 931 features (523 in positive and 408 in negative modes) with 102 common metabolites highly relevant to the presence of DR were detected. In the adjusted analysis, 67 metabolic features differed significantly between T2DM and NPDR patients. Pathway analysis revealed alterations in metabolisms of amino acids and fatty acids. Glutamate, phosphatidylcholine, and 13-hydroperoxyoctadeca-9,11-dienoic acid (13-PHODE) were key contributors to these pathway differences. A total of 171 features distinguished PDR patients from T2DM patients, and pathway analysis revealed alterations in amino acid metabolism, fatty acid metabolism, nitrogen metabolism, and tricarboxylic acid cycle. Aspartate, glutamate, glutamine, ornithine, N-acetyl-l-glutamate, N-acetyl-l-aspartate, citrate, succinate, N-(L-arginino)succinate, 2-oxoglutarate, 13-hydroperoxyoctadeca-9,11-dienoic acid, methionine, lysine, threonine, phenylalanine, N(pi)-methyl-l-histidine, phosphatidylcholine, and linoleate were major contributors to the pathway differences. Between NPDR patients and PDR patients, there were 79 significant differential metabolites. Enrichment pathway analysis showed changes in amino acid metabolism, fatty acid metabolism, pantothenate, and CoA biosynthesis. Aspartate, glutamine, N-acetyl-l-glutamate, N-acetyl-l-aspartate, pantothenate, dihomo-gamma-linolenate, docosahexaenoic acid, and icosapentaenoic acid were key factors for the differences of these pathways.

**Conclusion:** This study demonstrated that the pathways of arginine biosynthesis metabolism, linoleic acid metabolism, alanine, aspartate, and glutamate metabolism, as well as d-glutamine and d-glutamate metabolism, were dysregulated in DR patients of the Asian population. Increased levels of glutamate, aspartate, glutamine, N-acetyl-l-glutamate, and N-acetyl-l-aspartate and decreased levels of dihomo-gamma-linolenate, docosahexaenoic, and icosapentaenoic were considered as the metabolic profile that could distinguish PDR from NPDR in Asians. Phosphatidylcholine and 13-PHODE were identified as two major novel metabolite markers in advanced stages of DR in our study.

## Introduction

Diabetes is a prevalent global health problem that currently affects 537 million patients around the world (Sun et al., 2022). Without appropriate intervention, this number is projected to reach 643 million by 2030. Diabetes could lead to serious and even life-threatening complications, most notably cardiovascular diseases, diabetic nephropathy, and diabetic retinopathy. These complications could increase medical and nursing needs, reduce the quality of life of the patients, and substantially increase health care costs (Ogurtsova et al., 2017). Diabetic retinopathy (DR) is the most serious microvascular complication of diabetes in the eyes (Sumarriva et al., 2019) (Cabrera et al., 2020). It is also the main cause of decreased vision and blindness among 20–74-year-old adults in developing and developed countries (Sabanayagam et al., 2019). While enabling the increase in life expectancy, advances in modern medicine lead to an aging population and rising numbers of diabetic patients with complications such as diabetic retinopathy worldwide, especially in Asia (Yang et al., 2019) (Wong et al., 2016). The prevalence of diabetic retinopathy in China was estimated to be 1.14% (Zhong et al., 2018) (Song et al., 2018). As a destructive and progressive disease, DR can be divided into two stages according to its severity: non-proliferative diabetic retinopathy (NPDR) and proliferative diabetic retinopathy (PDR) (2016) (Mahajan et al., 2019). Currently, no treatment strategy for DR can consistently control the progression of each DR patient. Studies have shown that systemic risk factors of DR include higher levels of blood glucose, blood lipids, blood pressure, and longer disease course of diabetes (Sumarriva et al., 2019) (Cabrera et al., 2020), though studies have shown that higher blood glucose level and longer disease course of diabetes are the main risk factors that should be considered in clinical settings (Jenkins et al., 2015) (Cheung et al., 2014). However, these two factors alone could not completely predict an individual’s risk for developing diabetic retinopathy. In clinical practice, patients with similar durations of diabetes and similar levels of glycemic control could have tremendously different clinical outcomes in diabetic retinopathy. Some patients develop very mild retinopathy, while others seem to be significantly predisposed to severe retinopathy (Sun et al., 2011) (Gao et al., 2014) (Touzani et al., 2019). This phenomenon indicates that there may be other potential pathogenic factors behind DR and that there is still a lack of effective markers to detect and control the occurrence and progression of DR (Porta and Striglia, 2020). Therefore, it is of great importance to search for potential biomarkers that can predict the occurrence and development of DR, enabling better prevention and control of DR (Ting et al., 2016; Peng et al., 2018). Although there have been many metabolomic studies on DR, the identification of differential metabolites in critical periods of DR development (T2DM and NPDR periods) has been rarely attempted, especially in Asian populations. To address this gap and better understand the complicated metabolic state of DR, we performed untargeted metabolomics *via* LCMS in sera of the Asian patients with T2DM with and without DR. This study aimed to identify metabolites or metabolic pathways altered in DR in the Asian, as well as metabolic differences between patients with NPDR and PDR, especially the stage from T2DM to DR. Furthermore, we also compared the differential metabolism between DR group (including NPDR and PDR patients) and T2DM group as well as DR group and non-DR group (including T2DM and control patients). Identifying these differences in metabolic profiles could aid in the clarification of molecular mechanisms of DR and PDR and facilitate the identification of new therapeutic targets.

## Materials and Methods

### Ethics Statement

This study adhered to the tenets of the Declaration of Helsinki and was approved by the Ethical Committee of Peking University People’s Hospital. Signed informed consent was obtained from all participants.

### Study Participants and Research Design

This prospective observational registration study was conducted from December 2020 to July 2021 at Peking University People’s Hospital Ophthalmologic Center. A total of 618 patients with type 2 diabetes were screened. A cohort of 60 patients was randomly recruited from Peking University People’s Hospital Ophthalmologic Center. The diabetic control (*n* = 15) were healthy individuals with no history of diabetes. The T2DM cases (*n* = 15) included patients diagnosed with T2DM for at least 10 years with no clinical signs of DR as determined by dilated fundus examination by a retina specialist. DR cases (*n* = 30) were patients with T2DM and DR diagnosed on dilated fundus examination by a retina specialist. In accordance with the Early Treatment Diabetic Retinopathy Study (ETDRS) criteria, DR was classified into three categories: no DR, non-proliferative diabetic retinopathy (NPDR), and proliferative diabetic retinopathy (PDR) (Wilkinson et al., 2003) (Wu, 2013). All patients underwent thorough ophthalmic examination, color fundus photography, and optical coherence tomography (OCT). Color fundus photography and fluorescein angiography (FA) were obtained with FF 540 Plus (Carl Zeiss Meditech, Jena, Germany) or Optos 200Tx (Optos plc, Dunfermline, Scotland, United Kingdom). Optical coherence tomography (OCT) was performed with RTVue XR Avanti (Optovue, Fremont, CA, United States) or Cirrus HD-OCT 5000 (Carl Zeiss Meditec Inc., Dublin, CA, United States). Classification of no DR, NPDR (*n* = 15), or PDR (*n* = 15) was done by retina specialists with dilated fundus examination with the aid of color fundus photography, FFA, and OCT. Two or more ophthalmologists classified the DR status based on the results of the exams to avoid potential diagnosis bias. If there were discordance between the specialists, an agreement on the final diagnosis by all parties would be reached by open arbitration. A diagnosis of NPDR was based on the presence of blot hemorrhages, microaneurysms, cotton-wool spots, or intraretinal microvascular abnormalities on dilated fundus examination and no evidence of active PDR or history of treatment for PDR. A PDR diagnosis was based on the presence of neovascularization on the iris or retina or documented history of PDR for which the patient has received treatment. Exclusion criteria included 1) presence or history of other eye diseases (retinal degeneration, glaucoma, active ocular inflammation, etc.) or history of intraocular surgery (vitreoretinal surgery, intravitreal injection, laser therapy, and trauma history); 2) cancer, infectious disease, hyperuricemia, inherited metabolic diseases, mental disorder, heart failure, severe hypertension (systolic blood pressure ≥180 mm Hg or diastolic blood pressure ≥110 mm Hg), acute myocardial infarction, pregnancy, liver disease, stroke or any other severe chronic systemic disease; 3) corneal and lens pathologies that prevent a clear view of the fundus.

### Baseline Test/Data Collection and Definitions

All participants’ demographic information and medical records, including sex, age, past medical history, current status of smoking and alcohol consumption, duration of diabetes, clinical and laboratory measurements, drugs used, and disease status, were obtained. Patients received a general physical examination that included blood pressure, height, and weight measurements. The body mass indexes (BMI) of all patients were calculated and recorded. Blood laboratory tests, including fasting plasma glucose (FPG), total cholesterol (TC), triglycerides (TG), high-density lipoprotein cholesterol (HDL-c), low-density lipoprotein cholesterol (LDL-c), serum creatinine (SCr), hemoglobin A1c (HbA1c), and blood urea nitrogen (BUN), were measured using standard automated assays and recorded in the electronic case report form. Medication records included insulin, metformin, and other antidiabetic agents; ACE inhibitors, angiotensin receptor blockers, and other antihypertensive drugs; and statins and other lipid-lowering drugs.

### Blood Collection and Preparation

After at least 8 h overnight fasting, 6 ml of venous blood samples were collected under complete aseptic precautions using a 21 or 23 G butterfly needle from each study participant with tubes and stored at 4°C. The serum was separated by centrifugation at 3,000 rpm for 10 min (4°C) within 30 min to separate serum from whole blood and then transferred into a 1.5 ml sterile tube to be stored at −80°C ultra-low temperature allowing the serum to freeze immediately. Well-trained professional technicians carried out further measurements.

### Untargeted Metabolomics Analysis

Ultra-high-performance liquid chromatography coupled with tandem mass spectrometry (UHPLC-MS/MS) analyses were performed using a Vanquish UHPLC system (Thermo Fisher, Germany) coupled with an Orbitrap Q Exactive™ HF mass spectrometer (Thermo Fisher, Germany) in Novogene Co., Ltd. (Beijing, China). Before the analysis, frozen serum samples (100 μL) were thawed, dissolved at 4°C, placed in the Eppendorf (EP) tubes, and resuspended with prechilled 80% methanol and 0.1% formic acid by a well vortex. The samples were incubated on ice for 5 min and centrifuged at 15,000 g at 4 °C for 20 min. Some of the supernatant was diluted to a final concentration containing 53% methanol by LC-MS grade water. The samples were subsequently transferred to a fresh EP tube and then centrifuged at 15,000 g at 4°C for 20 min. Finally, the supernatant was injected into the LC-MS/MS system for analysis [22]. Samples were injected onto a Hypesil Goldcolumn (100 × 2.1 mm, 1.9 μm) using a 17 min linear gradient at a flow rate of 0.2 ml/min. The eluents for the positive polarity mode were eluent A (0.1% FA in water) and eluent B (methanol), and the eluents for the negative polarity mode were eluent A (5 mM ammonium acetate, pH 9.0) and eluent B (methanol). The solvent gradient was set as follows: 2% B, 1.5 min; 2%–100% B, 12.0 min; 100% B, 14.0 min; 100%−2% B, 14.1 min; and 2% B, 17 min. Q Exactive™ HF mass spectrometer was operated in positive/negative polarity mode with a spray voltage of 3.2 kV, capillary temperature of 320°C, sheath gas flow rate of 40 arb, and aux gas flow rate of 10 arb. Quality control (QC) samples were prepared by mixing the same amount of serum from each sample and using the same procedures as the test samples to extract metabolites. One QC was inserted into every 10 samples regularly before and after the operation. The raw data files generated by UHPLC-MS/MS were processed using the Compound Discoverer 3.1 (CD3.1, Thermo Fisher) to perform peak alignment, peak picking, and quantitation for each metabolite. The main parameters were set as follows: retention time tolerance, 0.2 min; actual mass tolerance, 5 ppm; signal intensity tolerance, 30%; signal/noise ratio, 3; and minimum intensity, 100,000. Peak intensities were normalized to the total spectral intensity. Based on additive ions, molecular ion peaks, and fragment ions, the normalized data were used to predict the molecular formula. Peaks were matched with the mzCloud, mzVault, and MassList database to obtain accurate qualitative and relative quantitative results.

### Data Processing and Pathway Analysis for Metabolomic Study

Statistical analyses were performed using the statistical software R (R version R-3.4.3), Python (Python 2.7.6 version), and CentOS (CentOS release 6.6). Normal transformation of non-normally distributed data was done using the area normalization method. These metabolites were annotated using the KEGG, HMDB, and LIPIDMaps databases. Multivariate analysis, including partial least squares discriminant analysis (PLS-DA), was used to determine the distributions and performed at metaX (a flexible and comprehensive software for processing metabolomics data). Univariate analysis (*t*-test) was used to calculate the statistical significance (*p*-value). To select the metabolites responsible for these differences, variable importance in the projection (VIP), fold changes (FC), and *p*-value were mainly used. The VIP value is an important parameter for detecting potential biomarker candidates that reflects the correlation of the metabolites with different biological states. In our study, VIP values >1.0 of PLS–DAs were used. *p* < 0.05 was considered statistically significant when analyzing differences among means. For pairwise comparisons, adjusted *p*-values using the Bonferroni correction as calculated by statistical software R were used. Therefore, the adjusted *p*-value < 0.05 was still considered significant. The relative metabolite levels were converted into fold changes (FC), which was defined as the ratio of each metabolite to the mean of all biological repeat quantitative values between groups. FC > 1.2 and < 0.833 indicated the significantly upregulated and downregulated differential metabolites, respectively. Volcano plots were calculated using filter metabolites of interest, based on log2 (FC) and −log10 (*p*-value) of metabolites, and the peaks that exhibited a statistically significant difference between two groups were used to perform multivariate pattern recognition. For clustering heat maps, the data were normalized using z-scores of the intensity areas of differential metabolites and plotted by the Pheatmap package in R language. The correlation between differential metabolites was analyzed by cor () in R language (method = Pearson). Statistically significant correlations between differential metabolites were calculated by cor.mtest () in R language. A *p*-value < 0.05 was considered statistically significant, and correlation plots were plotted by the corrplot package in R language. The functions of these metabolites and metabolic pathways were studied using the KEGG database. The metabolic pathway enrichment analysis of differential metabolites was performed when ratios were satisfied by x/n > y/N; metabolic pathways were considered enrichment when the p-value of metabolic pathway <0.05; and metabolic pathways were considered statistically significant enrichment.

### Bioinformatics Analysis

Descriptive statistics for demographic and clinical variables were calculated for the study population. Analysis of variance (ANOVA) was used to compare means of normally distributed data with homogeneity of variances. The Chi-square test was used for the analysis of categoric data (e.g., gender and presence of comorbidities). Wilcoxon rank-sum test was performed to compare age, diabetes duration, and biochemical parameters, including FPG, HbA1c, HDL-c, LDL-c, SCr, TC, TG, and BUN between the respective groups, as these values were not normally distributed within the groups.

## Results

### Baseline Characteristics

The demographic characteristics of the study population are shown in Table 1. Of all the participants (60 cases), 15 were T2DM patients (mean age of 73.53 ± 7.54 years, 40.0% males), 15 were NPDR patients (mean age of 70.47 ± 8.28 years, 60.0% males), 15 were PDR patients (mean age of 57.53 ± 9.05 years, 60.0% males), and 15 were controls (mean age of 64.07 ± 15.15 years, 46.7% males). Compared with T2DM patients and controls, PDR patients had a longer mean diabetes duration (*p* < 0.001, *p* < 0.001), but no differences were observed between NPDR and PDR, as well as T2DM and NPDR. The PDR group was significantly younger than the other groups (*p* = 0.028, *p* < 0.001, *p* < 0.001). No statistically significant differences in gender and body mass index (BMI) were found between groups. In the analysis of serum, fasting plasma glucose (*p* = 0.002, *p* = 0.001, *p* = 0.001) and HbA1c (*p* < 0.001, *p* < 0.001, *p* < 0.001) in control group was significantly lower than T2DM, NPDR, and PDR groups. Serum creatinine in the control, T2DM, and NPDR groups were significantly lower than in the PDR group (*p* = 0.045, *p* = 0.012, *p* = 0.032). Blood urea nitrogen in PDR patients was significantly higher than the other groups (*p* = 0.001, *p* < 0.001, *p* = 0.001). No statistically significant differences were found in levels of HDL-c, LDL-c, total cholesterol, triacylglycerol, and rate of hypertension between groups (Table 1).

**TABLE 1:** Demographics, comorbidities, and serum test results across groups.

	Control	T2DM	NPDR	PDR	P-value	P^a^	P^b^	P^c^	P^d^	P^e^	P^f^
Age	64.07 ± 15.15	73.53 ± 7.54	70.47 ± 8.28	57.53 ± 9.05	<0.001	0.082	0.209	0.028	0.965	<0.001	<0.001
Mean ± SD											
Gender, n (%)											
Male	7 (46.7)	6 (40)	9 (60)	9 (60)	0.615						
Female	8 (53.3)	9 (60)	6 (40)	6 (40)							
BMI	23.79 ± 3.26	23.90 ± 3.04	24.78 ± 3.90	24.65 ± 2.57	0.793	1.000	0.849	0.897	0.888	0.928	1.000
Diabetes duration, y	0	12.47 ± 2.94	16.67 ± 6.97	21.2 ± 6.10	<0.001	<0.001	<0.001	<0.001	0.113	<0.001	0.076
FPG (mm/L)	5.39 ± 0.66	7.89 ± 0.94	7.96 ± 1.65	8.06 ± 2.75	<0.001	0.002	0.001	0.001	1.000	0.994	0.999
HbA1 (mm/L)	5.13 ± 0.54	7.01 ± 0.67	7.21 ± 1.14	7.47 ± 0.93	<0.001	<0.001	<0.001	<0.001	0.925	0.501	0.861
HDL-c (mm/L)	1.35 ± 0.28	1.31 ± 0.22	1.24 ± 0.27	1.11 ± 0.17	0.057	0.965	0.625	0.051	0.884	0.145	0.483
LDL-c (mm/L)	2.95 ± 0.83	2.94 ± 0.98	3.06 ± 1.17	2.67 ± 0.74	0.724	>1.000	0.992	0.849	0.987	0.870	0.691
SCr (mm/L)	79.56 ± 17.83	71.33 ± 15.88	77.27 ± 15.47	122.40 ± 79.39	0.0082	0.955	0.999	0.045	0.982	0.012	0.032
TC (mm/L)	4.84 ± 0.94	4.81 ± 1.19	4.86 ± 1.41	5.03 ± 1.50	0.977	0.975	0.989	0.999	1.000	0.994	0.999
TG (mm/L)	1.26 ± 0.79	1.45 ± 0.70	1.79 ± 1.14	2.38 ± 2.43	0.196	0.984	0.762	0.181	0.926	0.337	0.707
BUN (mm/L)	5.55 ± 1.47	5.01 ± 1.31	5.64 ± 1.5	9.64 ± 4.80	<0.001	0.953	1.000	0.001	0.928	<0.001	0.001
HTN%	60.0%	66.7%	60.0%	73.3%	0.848						

For age, diabetes duration, FPG, HbA1c, HDL-c, LDL-c, SCr, TC, TG, and BUN, the mean and standard deviations are presented and comparisons were made by the Wilcoxon rank-sum test. Gender and rates of comorbidities were compared by X2 test. HbA1c and creatinine levels were taken from the date closest to the date of the blood draw. BMI, body mass index; FPG, fasting plasma glucose; HbA1c, hemoglobin A1c; HDL-c, high-density lipoprotein-cholesterol; LDL-c, low-density lipoprotein-cholesterol; SCr, serum creatinine; TC, total cholesterol; TG, triglycerides; BUN, blood urea nitrogen; HTN, hypertension. P^a^, *p*-value of diabetic controls *versus* T2DM patients. P^b^, *p*-value of diabetic controls *versus* NPDR patients. P^c^, *p*-value of diabetic controls *versus* PDR patients. P^d^, *p*-value of T2DM patients *versus* NPDR patients. P^e^, *p*-value of T2DM patients *versus* PDR patients. P^f^, *p*-value of NPDR patients *versus* PDR patients.

### Metabolites Identification Analysis

**FIGURE 1:**
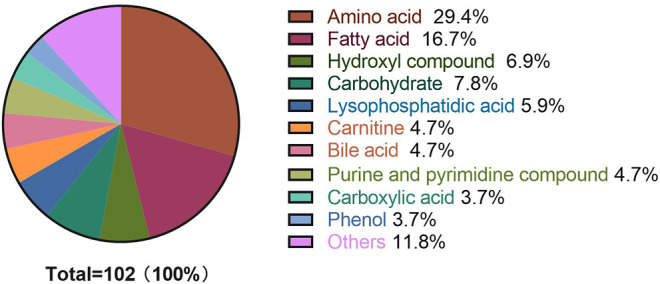
Metabolite classification analysis. The pie chart shows the 60 metabolites, including amino acids (29.4%), fatty acids (16.7%), hydroxyl compounds (6.9%), carbohydrates (7.8%), lysophosphatidic acids (5.9%), carnitines (4.7%), bile acids (4.7%), purine and pyrimidine compounds (4.7%) carboxylic acids (3.7%), phenols (3.7%), and others (11.8%). The highest percentage (30 out of 102) of these metabolites were amino acids.

**FIGURE 2:**
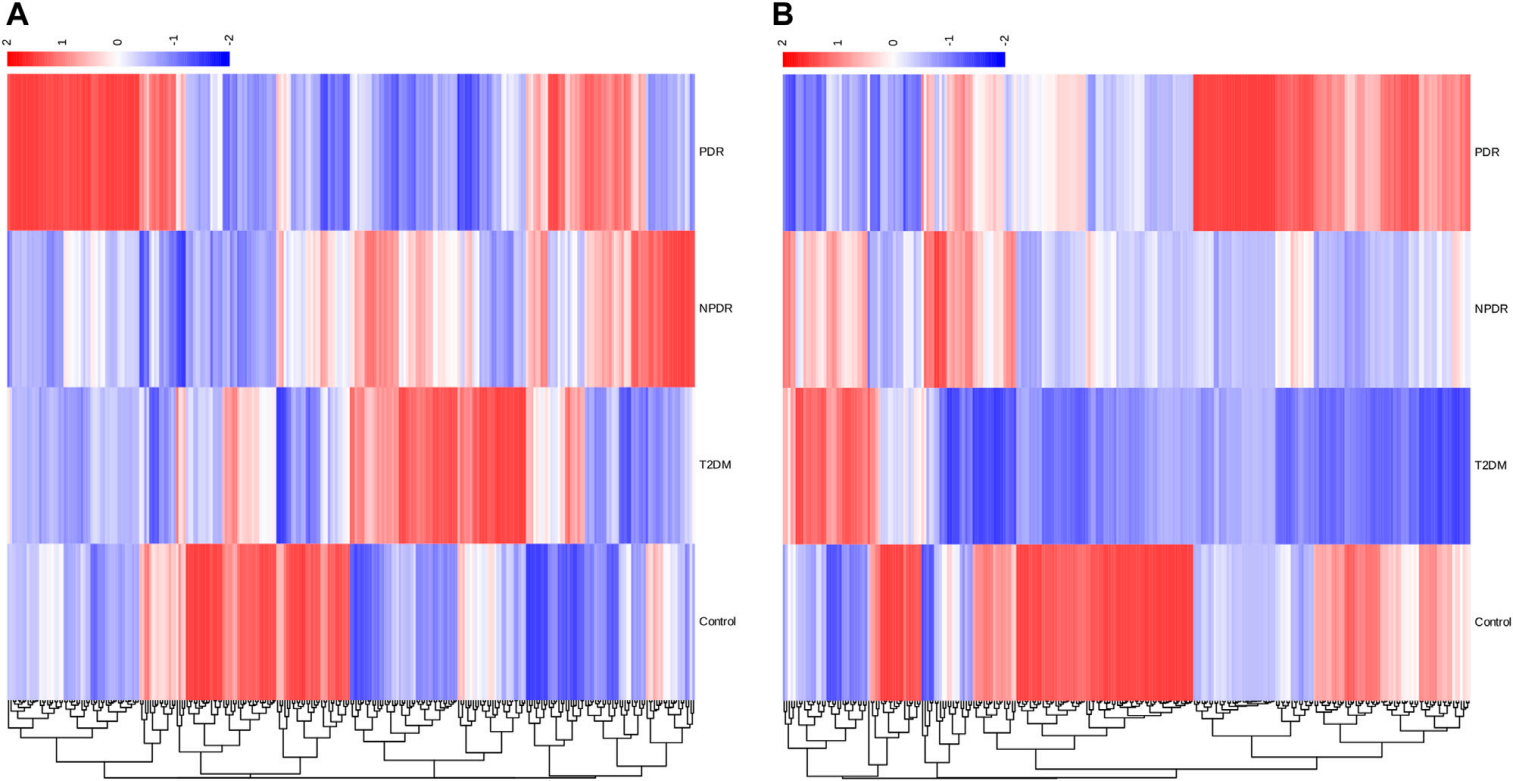
**(A)** Positive mode. **(B)** Negative mode. Hierarchical clustering analysis (HCA) showed that the identified metabolites were clearly grouped into controls, T2DM, and PDR patient clusters with high repeatability, and the resulting data were reliable and logical. Significant metabolic features increased (red) or decreased (blue) compared with the others group.

A metabolome-wide association study (MWAS) was performed to determine which metabolic features differed between control (*n* = 15), T2DM patients (*n* = 15), NPDR patients (*n* = 15), and PDR patients (*n* = 15) in the Asian population. In addition, we also conducted a further comparative analysis of metabolomics between the DR group (NPDR and PDR groups) and T2DM group, as well as between the DR group (NPDR and PDR groups) and non-DR group (T2DM and control groups). Through untargeted metabolomics analysis, 931 metabolic features (including 523 features in positive mode and 408 features in negative mode) were detected. A total of 102 common metabolites were identified by comparing MS fragment patterns with commercial standard compounds, and various databases, including the Kyoto Encyclopedia of Genes and Genomes (KEGG), Human Metabolome Database (HMDB), and LIPIDMaps databases, were selected. These metabolites were identified as metabolites that differed significantly among the groups, including 30 amino acids, 7 hydroxyl compounds, 4 carboxylic acids, 5 carnitines, 17 fatty acids, 5 bile acids, 8 carbohydrates, 5 purine and pyrimidine compounds, 3 phenols, and 6 lysophosphatidic acids. Detailed information on these metabolites is presented in Figure 1. Hierarchical clustering analysis (HCA) showed the relationship between the metabolite content clustering between groups. In both positive and negative modes, the identified metabolites in the controls, T2DM, and PDR groups showed distinguishable clusters in groups, even though the sample clusters overlapped slightly (Figure 2).

### Non-Proliferative DR *Versus* Type 2 Diabetic Mellitus

**FIGURE 3:**
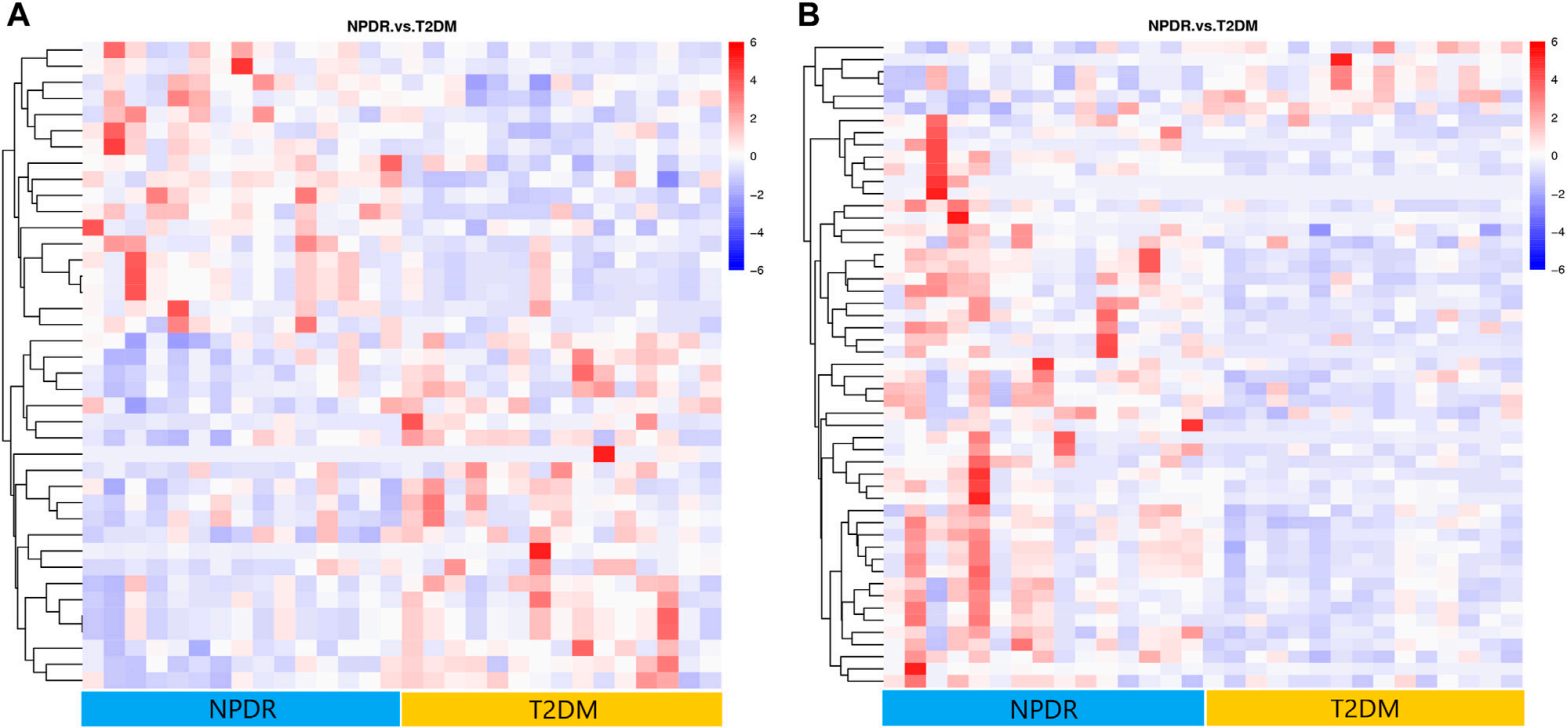
**(A)** Positive mode. **(B)** Negative mode. Hierarchical clustering analysis based on the intensity of significant metabolite features selected by PLS-DA identified clusters of features that were increased (red) or decreased (blue) in NPDR and T2DM patients. There are 40 metabolic features shown in positive mode and 53 metabolic features shown in negative mode.

**FIGURE 4:**
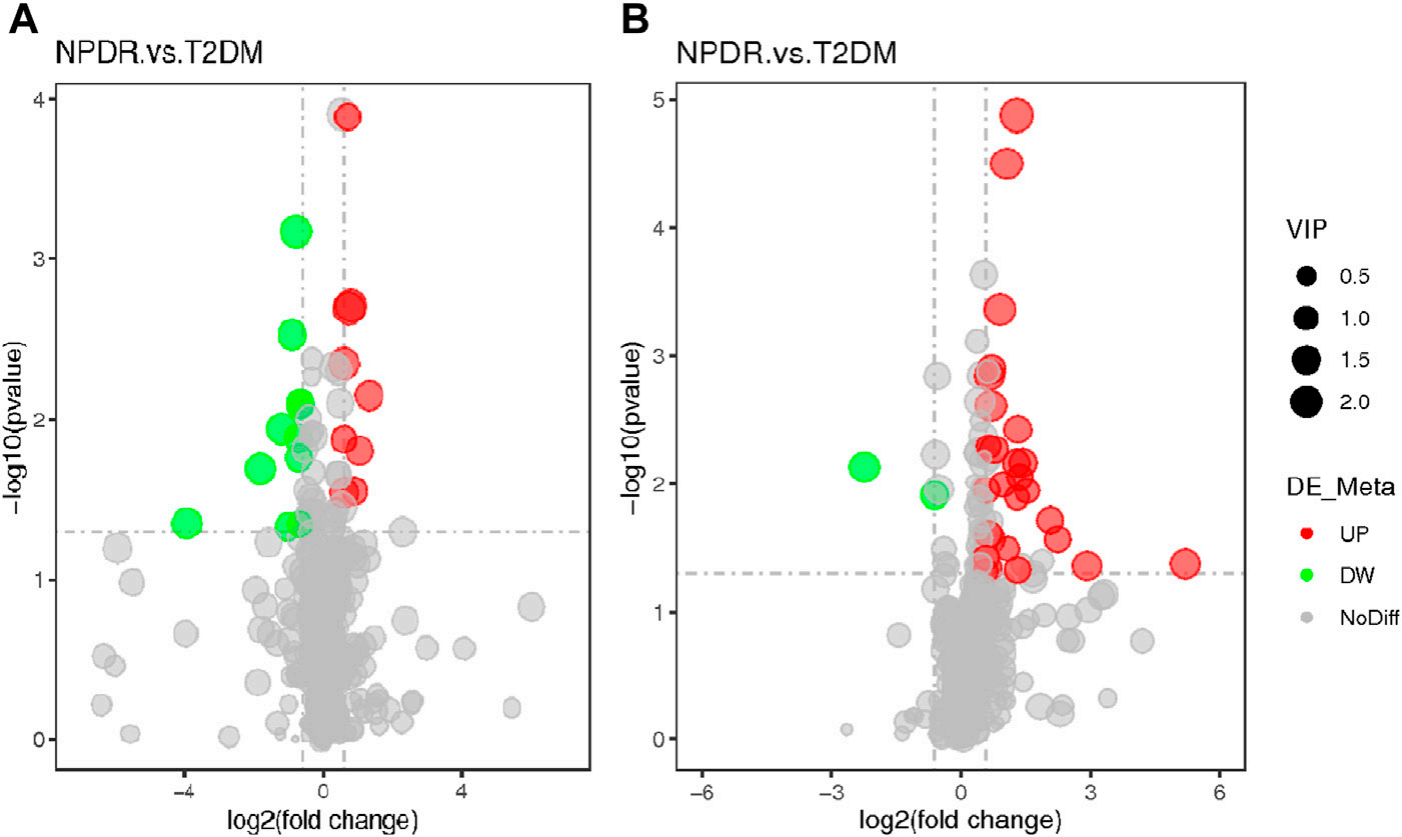
**(A)** Positive mode. **(B)** Negative mode. The volcano map of the log2 (FC) and −log10 (*p*-value) shows that 93 features were significantly different between NPDR patients (*n* = 15) and T2DM patients (*n* = 15). Significant metabolic features that increased (red dots) or decreased (green dots) in NPDR patients compared with T2DM patients are indicated, and the dot size represents the VIP value. In the positive mode, compared with T2DM patients, 12 metabolic features increased significantly and 14 metabolic features decreased significantly in NPDR patients. In the negative mode, compared with T2DM patients, 35 metabolic features increased significantly and 6 metabolic features decreased significantly in NPDR patients.

**FIGURE 5:**
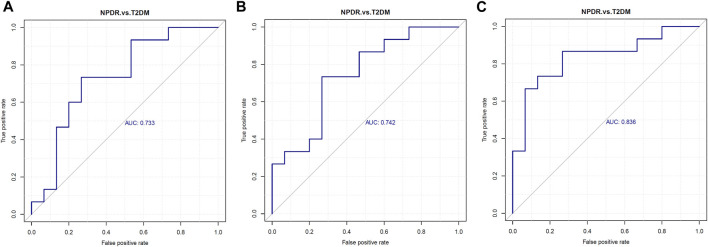
Serum levels of **(A)** glutamate, **(B)** phosphatidylcholine, and **(C)** 13-hydroperoxyoctadeca-9,11-dienoic acid were elevated in NPDR patients. Metabolites enriched in the pathway analyses were further analyzed with *p* ≤ 0.05 and using LC-MS/MS, revealing glutamate (*p* = 0.036, AUC = 0.733, VIP = 1.054), phosphatidylcholine (*p* = 0.022, AUC = 0.740,VIP = 1.581), and 13-hydroperoxyoctadeca-9,11-dienoic acid (*p* = 0.002, AUC = 0.835, VIP = 2.622) levels were significantly increased in NPDR patients compared with T2DM patients. AUC, area under the curve.

Based on PLS-DA and adjusted linear regression analysis (*p* < 0.05), there were 93 (40 in positive and 53 in negative modes) metabolic features that differed significantly between NPDR and T2DM patients in the adjusted analysis. These features were compared using hierarchical clustering analysis (Figure 3). The volcano maps visualized the differences between NPDR and T2DM patients in positive and negative modes based on log2 (FC) and −log10 (*p*-value) of metabolites (Figure 4). Excluding the metabolic features that could not be matched in mzCloud, mzVault, or MassList databases, 26 and 41 of the metabolites showed good discriminatory power for NPDR *versus* T2DM subjects with an area under the curve (95% CI) > 0.7 in positive and negative modes. We performed KEGG enrichment pathway analysis with VIP > 1.0 and AUC > 0.7 using the 67 features distinguishing NPDR patients and T2DM patients. This revealed enrichment of 22 metabolic pathways, three of which (metabolism of d-glutamine and d-glutamate, linoleic acid, and nitrogen) were considered to be the significant KEGG enrichment pathways with a *p*-value ≤ 0.05. The other pathways, including arginine biosynthesis, primary bile acid biosynthesis, metabolism of alpha-linolenic acid metabolism, and histidine, are shown in Table 2 and Supplementary Figure S1. Metabolites that were key contributors to the pathway differences confirmed with the metabolomics standards initiative (MSI) including glutamate (*p* = 0.036, AUC = 0.733), phosphatidylcholine (*p* = 0.022, AUC = 0.740), and 13-hydroperoxyoctadeca-9,11-dienoic acid (13-HPODE) (*p* = 0.002, AUC = 0.835) showed marked increase in NPDR subjects (Figure 5).

**TABLE 2:** KEGG enrichment pathways altered in NPDR patients compared with T2DM patients.

Pathway	Overlapping features	Pathway size	*p*-value
Linoleic acid metabolism	2	5	0.002
d-Glutamine and d-glutamate metabolism	1	6	0.038
Nitrogen metabolism	1	6	0.038
Arginine biosynthesis	1	14	0.204
primary bile acid biosynthesis	1	46	0.168
Alpha-linolenic acid metabolism	1	13	0.191
Histidine metabolism	1	16	0.230

With *p*-value ≤0.05, three metabolic pathways of linoleic acid (*p* = 0.0024), d-glutamine and d-glutamate (*p* = 0.0381), and nitrogen (*p* = 0.0381) were considered to be the significant KEGG enrichment pathways. Overlapping features represent the number of metabolites enriched in the pathway, while pathway size describes the total number of metabolites in each pathway.

### Proliferative DR *Versus* Type 2 Diabetic Mellitus

**FIGURE 6:**
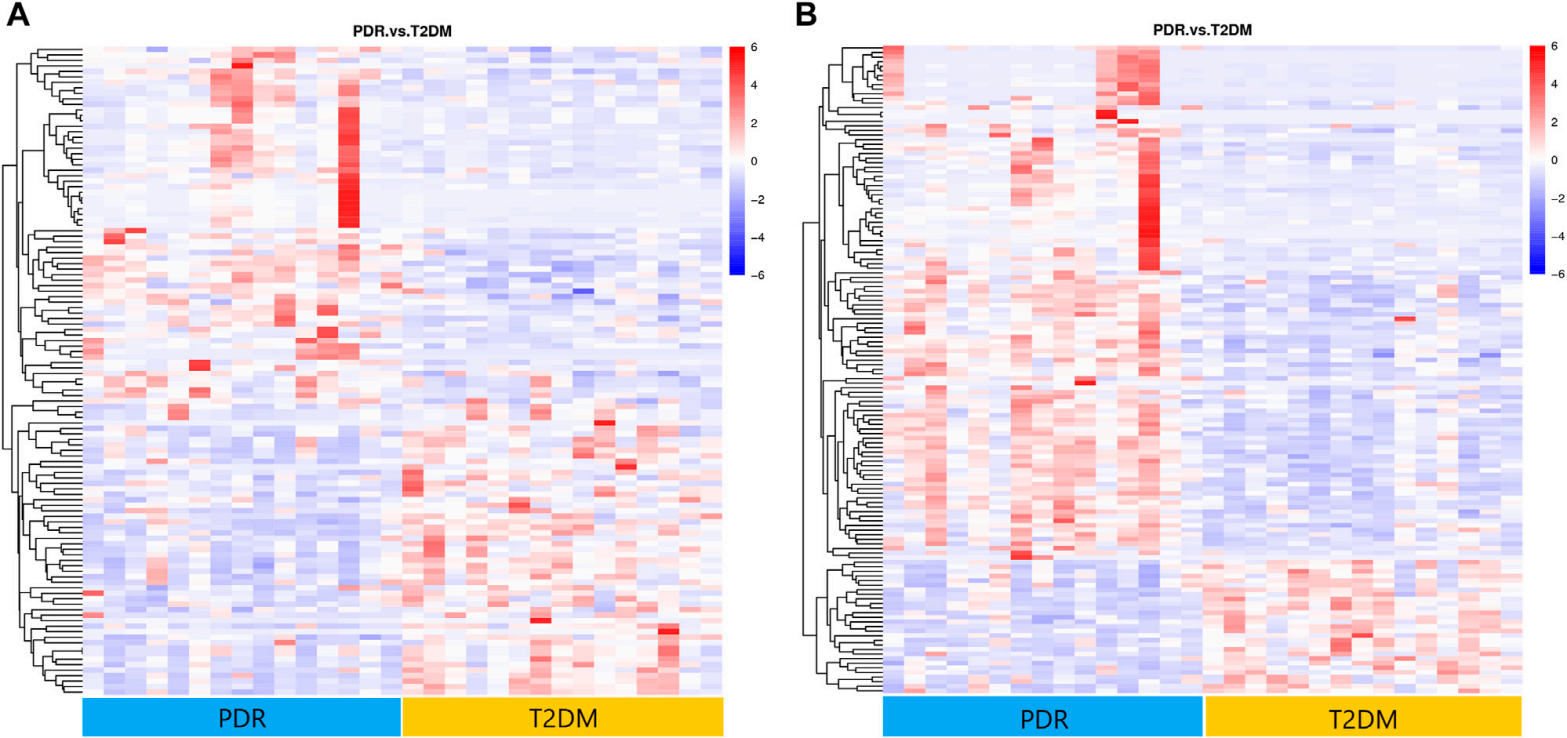
**(A)** Positive mode. **(B)** Negative mode. Hierarchical clustering analysis based on the intensity of significant metabolite features selected by PLS-DA identified clusters of features that were increased (red) or decreased (blue) in PDR and T2DM patients. There were 141 metabolic features shown in positive mode and 118 metabolic features shown in negative mode.

**FIGURE 7:**
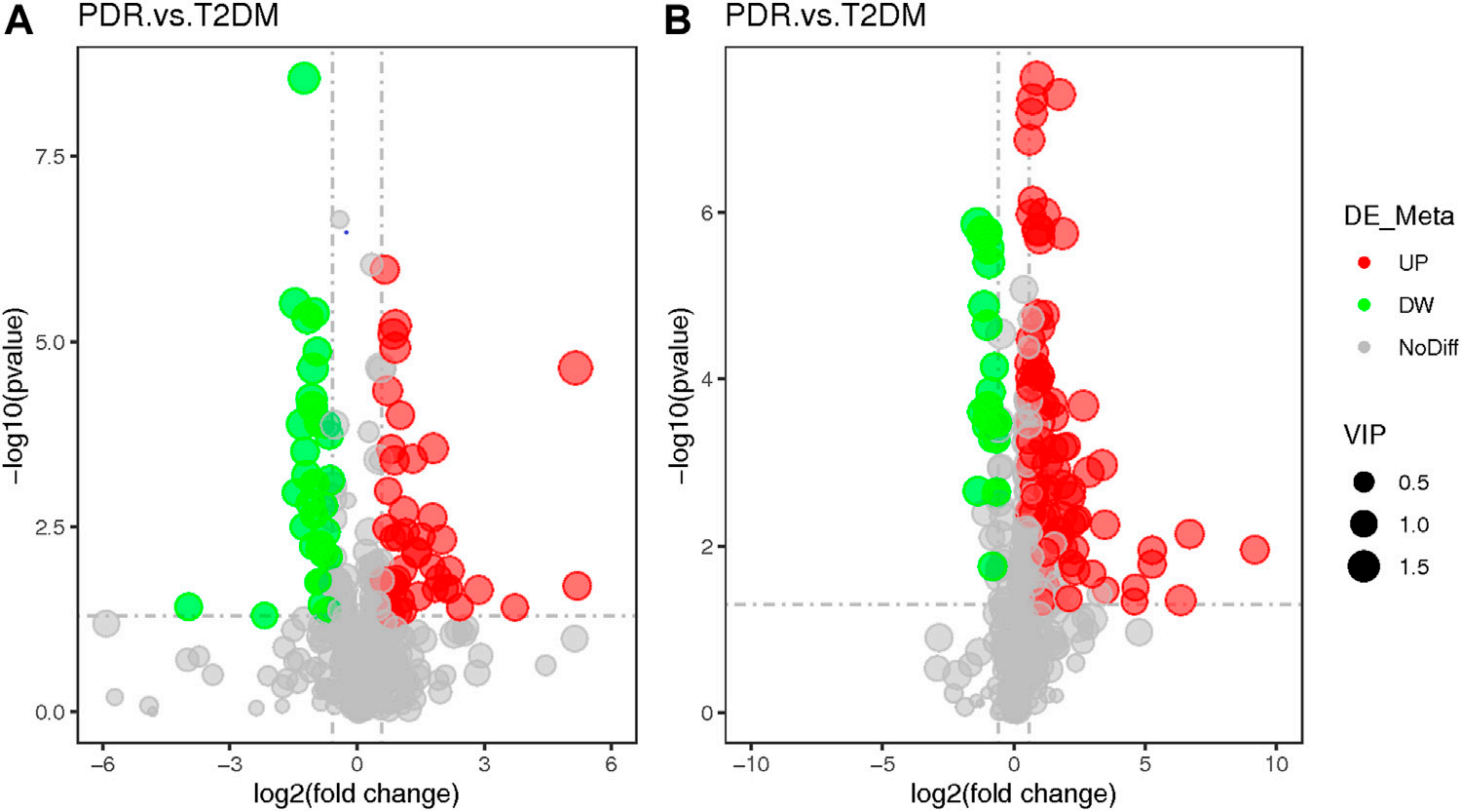
**(A)** Positive mode. **(B)** Negative mode. The volcano map of the log2 (FC) and −log10 (*p*-value) showed that 259 features were significantly different between PDR patients (*n* = 15) and T2DM patients (*n* = 15). Significant metabolic features that increased (red dots) or decreased (green dots) in PDR patients compared with T2DM patients are indicated with dot size representing the VIP value. In the positive mode, compared with T2DM patients, 39 metabolic features were significantly increased and 24 metabolic features were significantly decreased in PDR patients. In the negative mode, compared with T2DM patients, there were 82 metabolic features.

To determine metabolic features that differed between PDR and T2DM, an MWAS was performed to compare PDR patients (*n* = 15) with T2DM patients (*n* = 15). In the adjusted analysis, 259 features distinguishing PDR and T2DM groups were identified based on PLS-DA with a VIP >1.0 and adjusted linear regression analysis (*p* < 0.05). Hierarchical clustering analysis showed that between and within T2DM and NPDR groups, there were 118 and 141 metabolic features in positive and negative modes, respectively (Figure 6). Based on log2 (FC) and −log10 (*p*-value) of metabolites, the volcano maps visualized the differences between PDR patients and T2DM patients in positive and negative modes (Figure 7). In positive and negative modes, 63 and 108 of the metabolites, respectively, showed good discriminatory power for PDR *versus* T2DM subjects, with an area under the curve (95% CI) > 0.7. In KEGG enrichment pathway analysis, 171 features between PDR patients and T2DM patients revealed enrichment of 39 metabolic pathways, nine of which (metabolism of arginine biosynthesis, linoleic acid, alanine, aspartate and glutamate, d-glutamine and d-glutamate, aminoacyl-tRNA biosynthesis, butanoate, nitrogen, histidine, and tricarboxylic acid cycle) were considered to be the significant KEGG enrichment pathways with *p*-value ≤ 0.05. The other pathways included biosynthesis of unsaturated fatty acids, glutathione metabolism and glyoxylate and dicarboxylate metabolism (Table 3 and Supplementary Figure S2). Compared with T2DM, metabolites that were major contributors to the pathway differences were increased markedly in PDR subjects, including aspartate (*p* = 0.001, AUC = 0.996), glutamate (*p* = 1.70E-05, AUC = 0.916), glutamine (*p* = 6.43E-05, AUC = 0.876), ornithine (*p* = 9.50E-04, AUC = 0.827), 2-oxoglutarate (*p* = 0.007, AUC = 0.822), N-acetyl-l-glutamate (*p* = 0.002, AUC = 0.871), N-acetyl-l-aspartate (*p* = 0.004, AUC = 0.813), citrate (*p* = 0.011, AUC = 0.813), phosphatidylcholine (*p* = 0.001, AUC = 0.871), 13-HPODE (*p* = 0.001, AUC = 0.796), methionine (*p* = 0.001, AUC = 0.840), lysine (*p* = 0.001, AUC = 0.831), threonine (*p* = 0.002, AUC = 0.836), phenylalanine (*p* = 0.010, AUC = 0.751), N-(L-arginino) succinate (*p* = 0.026, AUC = 0.796), succinate (*p* = 0.030, AUC = 0.711), and N (pi)-methyl-l-histidine (*p* = 0.031, AUC = 0.716). On the contrary, linoleate level was (*p* = 5.31E-04, AUC = 0.871) significantly lower in PDR subjects (Supplementary Figure S3).

**TABLE 3:** KEGG enrichment pathways altered in PDR patients compared with T2DM patients.

Pathway	Overlapping features	Pathway size	*p*-value
Arginine biosynthesis	8	14	1.7 × 10−8
Alanine, aspartate, and glutamate metabolism	8	28	1.1 × 10−5
Linoleic acid metabolism	3	5	6.9 × 10−4
d-Glutamine and d-glutamate metabolism	3	6	1.3 × 10−3
Aminoacyl-tRNA biosynthesis	7	48	3.4 × 10−3
Butanoate metabolism	3	15	0.023
Nitrogen metabolism	2	6	0.024
Histidine metabolism	3	16	0.028
Tricarboxylic acid cycle	3	20	0.043

With *p*-value ≤ 0.05, nine metabolism pathways including arginine biosynthesis (*p* = 1.7 × 10−8), alanine, aspartate and glutamate (*p* = 1.1 × 10−5), linoleic acid (*p* = 6.9 × 10−4), d-glutamine and d-glutamate (*p* = 1.3 × 10−3), aminoacyl-tRNA, biosynthesis (*p* = 3.4 × 10−3), butanoate (*p* = 0.023), nitrogen (*p* = 0.024), histidine (*p* = 0.028), and tricarboxylic acid cycle (*p* = 0.049) were considered to be the significant KEGG enrichment pathways. Overlapping features represent the number of metabolites enriched in the pathway, while pathway size describes the total number of metabolites in each pathway.

### Proliferative DR *Versus* Non-Proliferative DR

**FIGURE 8:**
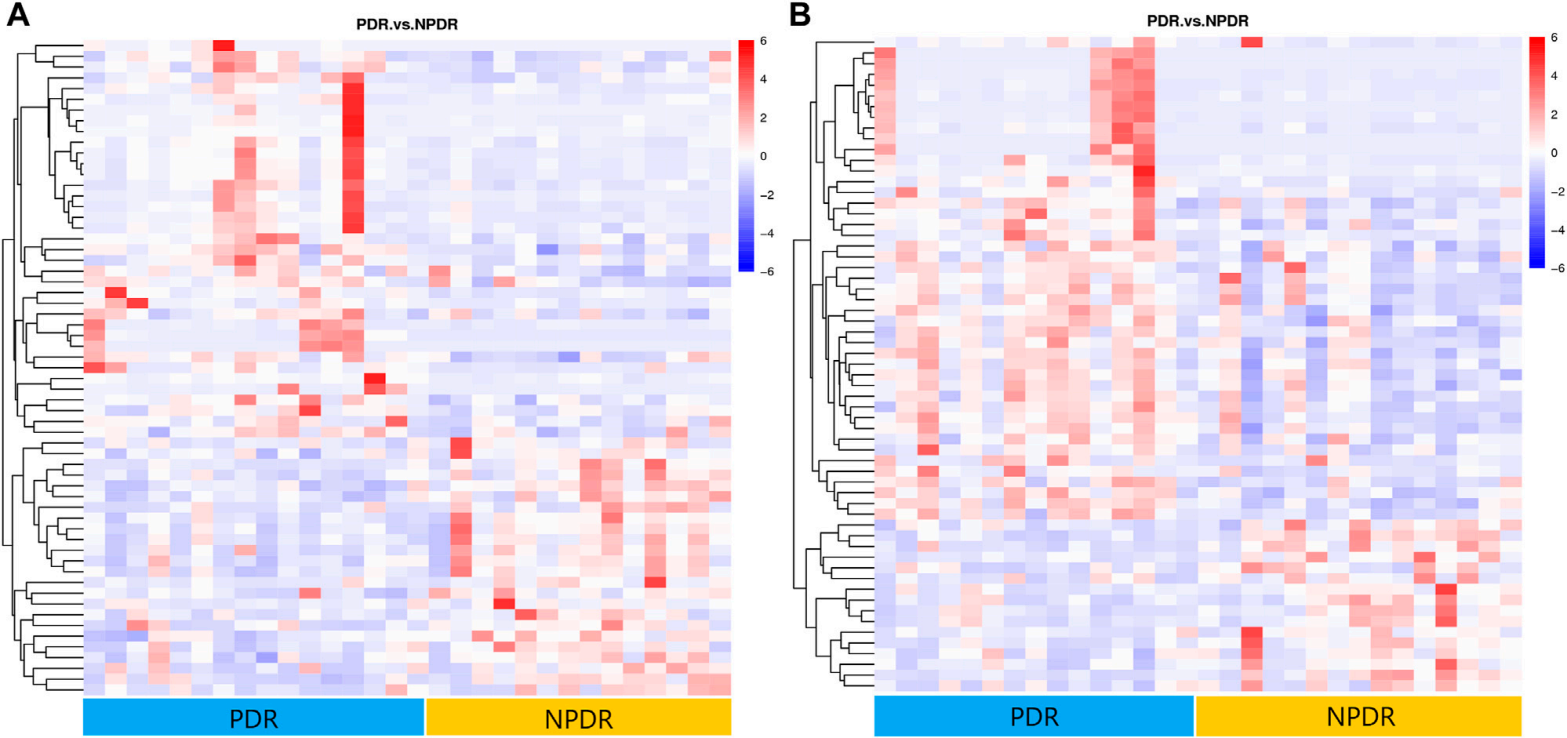
**(A)** Positive mode. **(B)** Negative mode. Hierarchical clustering analysis based on the intensity of significant metabolite features selected by PLS-DA with a VIP > 1.0 identified clusters of features that were increased (red) or decreased (blue) in PDR patients and NPDR patients. There are 61 metabolic features shown in positive mode and 61 metabolic features shown in negative mode.

**FIGURE 9:**
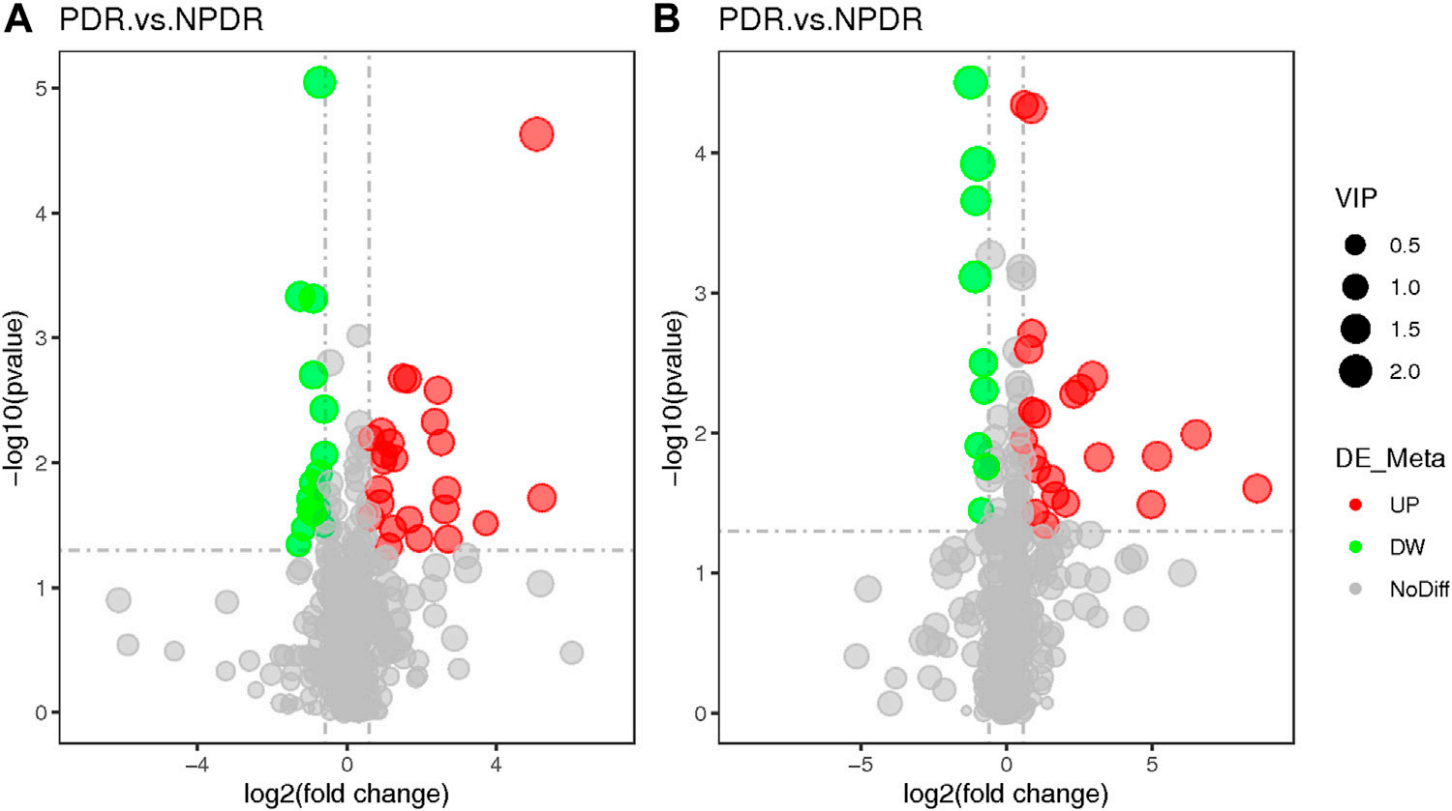
**(A)** Positive mode. **(B)** Negative mode. Based on log2 (FC) and −log10 (*p*-value) of metabolites, the volcano map showed that 122 features were significantly different between PDR patients (*n* = 15) and T2DM patients (*n* = 15). Metabolic features that were significantly higher (red dots) or lower (green dots) in PDR patients compared with NPDR patients were indicated, with dot size representing the VIP value. In the positive mode, compared with NPDR patients, 20 metabolic features were significantly increased and 11 metabolic features were significantly decreased in PDR patients. In the negative mode, compared with NPDR patients, 33 metabolic features were significantly increased and 15 metabolic features were significantly decreased in PDR patients.

A total of 122 metabolic distinguishable features were detected between PDR patients (*n* = 15) and NPDR patients (*n* = 15) based on PLS-DA with a VIP >1.0 and adjusted linear regression analysis (*p* < 0.05). There were 61 and 61 distinguishable metabolic features in positive and negative modes, respectively, in hierarchical clustering analysis (Figure 8). According to log2 (FC) and −log10 (*p*-value) of metabolites, the volcano map could more intuitively display the upregulation and downregulation of differential metabolites in positive and negative patterns between PDR patients and NPDR patients (Figure 9). In positive and negative modes with an area under the curve (95% CI) > 0.7, 31 and 48 of the metabolites, respectively, show good discriminatory power for PDR *versus* NPDR subjects. KEGG enrichment pathway analysis using the 79 differential metabolites with VIP > 1.0 and AUC > 0.7 identified enrichment in arginine biosynthesis metabolism, alanine, aspartate and glutamate metabolism, d-glutamine and d-glutamate metabolism, biosynthesis of unsaturated fatty acids, and pantothenate and CoA biosynthesis (Table 4 and Supplementary Figure S4). A Wilcoxon rank sum test was performed on the metabolites that contributed to the enrichment of these pathways to prioritize key metabolites. Compared with NPDR patients, in PDR patients, five key factors, including aspartate (*p* = 0.004, AUC = 0.853), glutamine (*p* = 6.73E-04, AUC = 0.840), N-acetyl-l-glutamate (*p* = 0.028, AUC = 0.702), N-acetyl-l-aspartate (*p* = 0.021, AUC = 0.707), and pantothenate (*p* = 0.025, AUC = 0.729), significantly increased, and three key factors, including dihomo-gamma-linolenate (*p* = 5.65E-04, AUC = 0.849), docosahexaenoic acid (*p* = 0.005, AUC = 0.809), and icosapentaenoic (*p* = 0.012, AUC = 0.747) levels, were significantly lower (Supplementary Figure S5).

**TABLE 4:** KEGG enrichment pathways altered in PDR patients compared with NPDR patients.

Pathway	Overlapping features	Pathway size	*p*-value
Arginine biosynthesis	4	14	1.1 × 10^−4^
Alanine, aspartate, and glutamate metabolism	5	28	1.6 × 10^−4^
d-Glutamine and d-glutamate metabolism	2	6	5.5 × 10^−3^
Biosynthesis of unsaturated fatty acids	3	36	0.033
Pantothenate and CoA biosynthesis	2	19	0.043

With *p*-value ≤0.05 in the Wilcoxon rank-sum test, six metabolic pathways, including arginine biosynthesis metabolism (*p* = 1.1 × 10−4), alanine, aspartate and glutamate metabolism (*p* = 1.6 × 10−4), d-glutamine and d-glutamate metabolism (*p* = 5.5 × 10−3), biosynthesis of unsaturated fatty acids (*p* = 0.033), and pantothenate and CoA biosynthesis (*p* = 0.043), were considered to be the significant KEGG enrichment pathways. Overlapping features represent the number of metabolites enriched in the pathway, while pathway size describes the total number of metabolites in each pathway.

**FIGURE 10:**
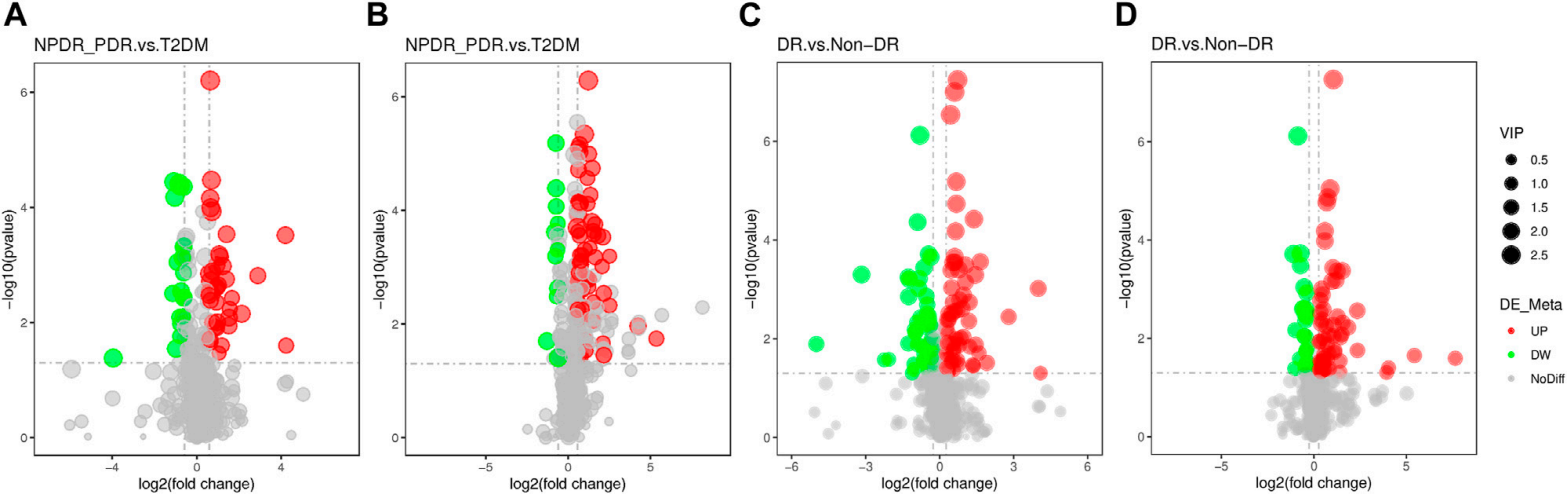
**(A)** DR (including NPDR and PDR) *versus* T2DM in the positive mode. **(B)** DR (including NPDR and PDR) *versus* T2DM in the negative mode. **(C)** DR *versus* non-DR in the positive mode. **(D)** DR *versus* non-DR in the negative mode. Metabolic features were significantly increased (red dots) or decreased (green dots) in the volcano maps based on log2 (FC) and −log10 (*p*-value) of metabolites. **(A)** and **(B)** The volcano map, respectively, showed that 96 and 113 features were significantly different in positive and negative modes between DR patients (*n* = 30) and T2DM patients (*n* = 15). **(C)** and **(D)** The volcano map, respectively, showed that 112 and 86 features were significantly different in positive and negative modes between DR patients (*n* = 30) and non-DR patients (*n* = 30).

We further grouped and analyzed the differential metabolites between DR patients (including NPDR and PDR patients) (*n* = 30) and T2DM patients (*n* = 15), as well as DR patients (including NPDR and PDR patients) (*n* = 30) and non-DR patients (including T2DM patients and diabetic controls) (*n* = 30). In the adjusted analysis based on PLS-DA and adjusted linear regression analysis (*p* < 0.05), 209 and 198 distinguishable metabolic features were detected in DR *versus* T2DM and DR *versus* non-DR, respectively. With an area under the curve (95% CI) > 0.7 in the volcano maps, 138 and 133 metabolites showed good discriminatory power for DR *versus* T2DM subjects and DR *versus* non-DR subjects, respectively (Figure10). In KEGG enrichment pathway analysis, the pathways that showed significant enrichment in both DR *versus* T2DM and DR *versus* non-DR were arginine biosynthesis, alanine, aspartate and glutamate metabolism, linoleic acid metabolism, and d-glutamine and d-glutamate metabolism (Table5 and Supplementary Figure S6). The other pathways included aminoacyl-tRNA biosynthesis (*p* = 0.005) and nitrogen metabolism (*p* = 0.017) in DR *versus* T2DM, and butanoate metabolism (*p* = 0.017) in DR *versus* non-DR. There were 12 key contributors to these pathway differences between DR patients and T2DM patients, 11 of which (glutamate, N-acetyl-l-glutamate, 13-HPODE, aspartate, N-(L-arginino) succinate, threonine, methionine, phosphatidylcholine, oxoglutarate, glutamine, and ornithine) were significantly increased in DR patients, while only the level of eicosatrienoic acid was decreased in DR patients. Compared with non-DR patients, six key factors (glutamate, aspartate, N-acetyl-l-glutamate, 13-HPODE, phosphatidylcholine, and 3-hydroxybutanoate) were significantly increased in DR patients.

**TABLE 5:** KEGG enrichment pathways altered in both DR *versus* T2DM and DR *versus* non-DR.

Pathway	DR *versus* T2DM		DR *versus* non-DR	
	Overlapping features/pathway size	*p*-value	Overlapping features/pathway size	*p*-value
Arginine biosynthesis	8/14	3.3 × 10^−9^	5/14	1.0 × 10^−4^
Alanine, aspartate, and glutamate metabolism	6/28	2.8 × 10^−4^	6/28	4.6 × 10^−4^
d-Glutamine and d-glutamate metabolism	3/6	7.4 × 10^−4^	2/6	0.019
Linoleic acid metabolism	2/5	0.011	2/5	0.013

There were four enrichment pathways indicated simultaneously in DR *versus* T2DM and DR *versus* non-DR in KEGG enrichment pathway analysis with *p*-value ≤0.05 in the Wilcoxon rank-sum test.

## Discussion

In order to identify complex endogenous metabolic phenotypes of multifactorial diseases, metabolomics is a powerful tool that can reflect the influence of pathological factors from different sources and the pathophysiological states of diseases (Alshehry et al., 2016) (Mapstone et al., 2014) (Zuo et al., 2021) (Baharum and Azizan, 2018) (Chen et al., 2016).

In our study, the metabolic pathways of arginine biosynthesis and alanine, aspartate, and glutamate metabolism were identified as the major specific metabolic pathways in PDR *versus* T2DM, PDR *versus* NPDR, DR *versus* T2DM, and DR *versus* non-DR. A similar result was observed in a vitreous humor liquid chromatography-mass spectrometry (LC-MS) based metabolomics study (Paris et al., 2016). Numerous studies on diabetic patients and a variety of experimental animal models have demonstrated the importance of the urea cycle and arginine metabolism in diabetes-induced oxidative stress, inflammation, and vascular dysfunction (Sumarriva et al., 2019) (Cao et al., 2019) (Caldwell et al., 2018). Arginine metabolism is a complex process. Ornithine, as a non-protein amino acid, is an intermediate in arginine-proline metabolism and a core part of the urea cycle (Tong et al., 2018). Consistent with our finding, Zuo et al. detected that the intensity of serum ornithine was positively linked to elevated odds of DR (Zuo et al., 2021). To our knowledge, the mechanism of ornithine in DR has not been examined so far. Ornithine is the substrate for the ornithine decarboxylase (ODC) pathway for the production of different polyamines through modulating ODC/polyamine systems. It is also a substrate for ornithine aminotransferase (OAT) to produce proline, which is a critical component for collagen formation (Morris, 2016). Under a hyperglycemic state, arginine produces ornithine and urea by arginine II enzyme (Arg-II enzyme). The elevation of ornithine level indicates the increased activity of the Arg-II enzyme, involved in microglia and macrophage-mediated chronic inflammation injury in type 2 diabetes (Narayanan et al., 2014). Meanwhile, the increased activity of the Arg-II enzyme declined the activity of the nitric oxide synthase (NOS) pathway, which mainly produces nitric oxide. Deficiency of nitric oxide and increased level of polyamine and proline could cause endothelial cell dysfunction, impaired vasodilation function, and inducement of cell proliferation and fibrosis (Stehouwer, 2018). We speculated that this might be closely related to the occurrence and development of DR. Meanwhile, the metabolic roles of serum glutamine, glutamate, and aspartate in diabetic retinopathy have not been clarified. A relevant study in 2021 reported that the pathogenesis of patients with impaired fasting glucose, diabetic microvascular complications, and diabetic peripheral vascular disease was highly correlated with serum glutamine, glutamate, and aspartate metabolism (Li et al., 2021). Another study reported similarly altered patterns of serum amino acids, including glutamine, asparagine, and aspartic acid, in diabetic patients compared with non-diabetic subjects (Zhou et al., 2013).

Another pathway highlighted in NPDR *versus* T2DM, PDR *versus* T2DM, and PDR *versus* NPDR comparisons was d-glutamine and d-glutamate metabolism. Glutamate, derived from glucose through the malate-aspartate shuttle, is not only the signal underlying incretin-induced insulin secretion (Gheni et al., 2014) but also the main excitatory neurotransmitter in the brain, spinal cord, and retina (Takahashi et al., 2019) (Liew et al., 2017) which further leads to the damage of oxidative tissue and increased level of free radicals (Du et al., 2018). Moreover, the ratio of glutamine to glutamate and serum glutamine concentration is closely related to various cellular functions and insulin resistance (Gheni et al., 2014) (Jenstad and Chaudhry, 2013) (Cheng et al., 2012). The roles of serum glutamine and glutamic acid in DR have not been elucidated yet. However, some evidence has pointed out that as diabetes progresses, the accumulation of glutamate in the retina will produce neurotoxic effects, generate an uncontrolled intracellular calcium response in postsynaptic neurons, and accelerate the development of DR and cell death (Coughlin et al., 2017) (Narayanan et al., 2019) (Lieth et al., 2000). In a serum-targeted metabolomics study of diabetic retinopathy, Rhee et al. indicated that serum levels of glutamine and glutamate and their ratios might be novel biomarkers for predicting DR in T2DM. Furthermore, glutamine and glutamate were considered the most unique metabolites in DR (Rhee et al., 2018). These results were consistent with our findings.

Normally, polyunsaturated fatty acids are highly expressed in the retina and regulate many biological processes, including nerve protection, regulation of vascular endothelial growth factor expression, inhibition of retinal neovascularization, prevention of pericyte loss caused by retinal vascular inflammation, and maintenance of retinal capillary structure and integrity (Sasaki et al., 2015). Studies on the relationship between polyunsaturated fatty acids and DR suggested that an elevated level of polyunsaturated fatty acids was negatively correlated with DR and was an independent protective factor for DR (Li et al., 2020) (Zuo et al., 2021). However, a metabolomics study on serum reported that no significant differences in linoleic acid and arachidonic acid intensities exist between DR patients and diabetic controls (Chen et al., 2016). It is not yet clear whether the reduction in polyunsaturated fatty acid composition in diabetic patients is due to increased lipid peroxidation or the changes in lipid synthesis or circulation (Augustine et al., 2020). In our study, linoleic acid metabolic pathways were highlighted by comparing between NPDR and T2DM, PDR and T2DM, DR and T2DM, and DR and non-DR, which have also been reported in other metabolomics research on DR (Zuo et al., 2021) (Li et al., 2020).

To our knowledge, this study was the first to confirm that the serum levels of phosphatidylcholine and 13-PHODE were closely related to the different stages of diabetic retinopathy of type 2 diabetes in the Asian population. In our study, phosphatidylcholine and 13-PHODE levels were significantly elevated in DR patients compared with T2DM and non-DR patients. Although there is no relevant study on the pathogenesis of phosphatidylcholine in diabetic retinopathy at present, a large prospective study from Harvard University found that higher phosphatidylcholine intake was associated with an increased risk of type 2 diabetes (Li et al., 2015). In addition, a study using targeted metabolomics to identify serum metabolites associated with type 2 diabetes indicated that metabolic alterations, including amino acids and cholinergic phospholipids, were highly associated with a risk of early type 2 diabetes (Floegel et al., 2013). However, in an untargeted serum lipidomic analysis of diabetic retinopathy of type 1 diabetes, 104 lipids, including phosphatidylcholine, of five major lipids were identified as negatively associated with DR grading (Curovic et al., 2020). This discrepancy with our results may owe to the types of diabetes and distinct nationalities of the participants. Although the mechanism of 13-PHODE in the pathogenesis of diabetic retinopathy has not been clarified so far, 13-PHODE has been found to induce inflammatory responses in animal models, which could significantly induce pro-inflammatory gene expression of TNF-α and MCP-1 *in vitro*, most notably in differentiated intestinal epithelial cells (Keewan et al., 2020). The possible involvement of 13-PHODE in mitochondrial dysfunction-related disorders has also been reported (Faizo et al., 2021). Further studies are needed to confirm our findings.

In terms of differential metabolites, in our study, compared with T2DM patients, glutamate, phosphatidylcholine, and 13-PHODE were significantly increased in NPDR patients. The presence of these metabolites may suggest that the manifestations of ocular lesions in diabetic patients could shift from the T2DM stage to the DR stage. Furthermore, increased serum levels of glutamate, aspartate, glutamine, N-acetyl-l-glutamate, and N-acetyl-l-aspartate and decreased levels of dihomo-gamma-linolenate, docosahexaenoic, and cosapentaenoic could be used as differential metabolites between PDR and NPDR patients. These findings suggested that diabetes patients with these differential metabolite profiles should be observed more closely to avoid progression to PDR. Our findings may provide new insights into the management of DR patients and the development of novel treatments for DR patients.

The cohort of DR patients and diabetic controls in the Asian population enrolled from the same institution with standardized sample collection and processing are strengths of the study. Although the generalizability of these results is not yet certain because patients enrolled in this study were all from the same geographic region, the main DR metabolic pathways found in our study were mostly consistent with previous serum metabolomics analyses of DR in western countries. In recent years, there have been four serum untargeted metabolomic studies of diabetic retinopathy, three of which were analyzed through LC-MS (Zuo et al., 2021; Sumarriva et al., 2019; Zhu et al., 2019) and one using gas chromatography-mass spectrometry (GC-MS) (Rhee et al., 2018). Untargeted high-resolution LC-MS, used in this study, is a sensitive technology affected by diet, lifestyle, drugs, and gender that can reflect the overall dynamic changes of all endogenous metabolites such as nucleic acids, proteins, lipids, and other small molecules in organisms. Demographic information and comorbidities of enrolled patients were recorded in detail to minimize the effects of these confounding factors. A previous study by Zhu et al. on serum untargeted metabolomics of patients with DR in China identified four differential metabolites, including fumaric acid, uridine, acetic acid, and cytidine, as potential biomarkers for PDR, and nine KEGG pathways that were significantly enriched in PDR patients compared with NPDR patients (Zhu et al., 2019). Our study further analyzed the differences between NPDR and T2DM patients, PDR and T2DM patients, and DR and non-DR patients. However, studies with a larger sample size and targeted metabolomics validation are needed to determine the identification of metabolites and prevent potential confounding factors from untargeted studies. To our knowledge, this untargeted metabolomics study was the first to specifically compare the metabolic profiles of PDR and NPDR in the Asian population and analyze the metabolic differences between DR and T2DM patients, as well as DR patients and non-DR patients in the Asian population. In conclusion, this metabolomics analysis demonstrates that arginine metabolism, linoleic acid metabolism alanine, aspartate and glutamate metabolism, and d-glutamine and d-glutamate metabolism are dysregulated in the development of DR in the Asian population. Further studies are needed to clarify the roles these pathways and differential metabolites play in the pathogenesis and progression of DR.

## Data Availability

The raw data supporting the conclusions of this article will be made available by the authors without undue reservation.
