# Supplementary figures and images for: Serum Untargeted Metabolomics Reveal Potential Biomarkers of Progression of Diabetic Retinopathy in Asians
Source: Front Mol Biosci. 2022 Jun 9;9:871291. doi: 10.3389/fmolb.2022.871291 (PMC9224596; doi:10.3389/fmolb.2022.871291)

**PDR.vs.NPDR**

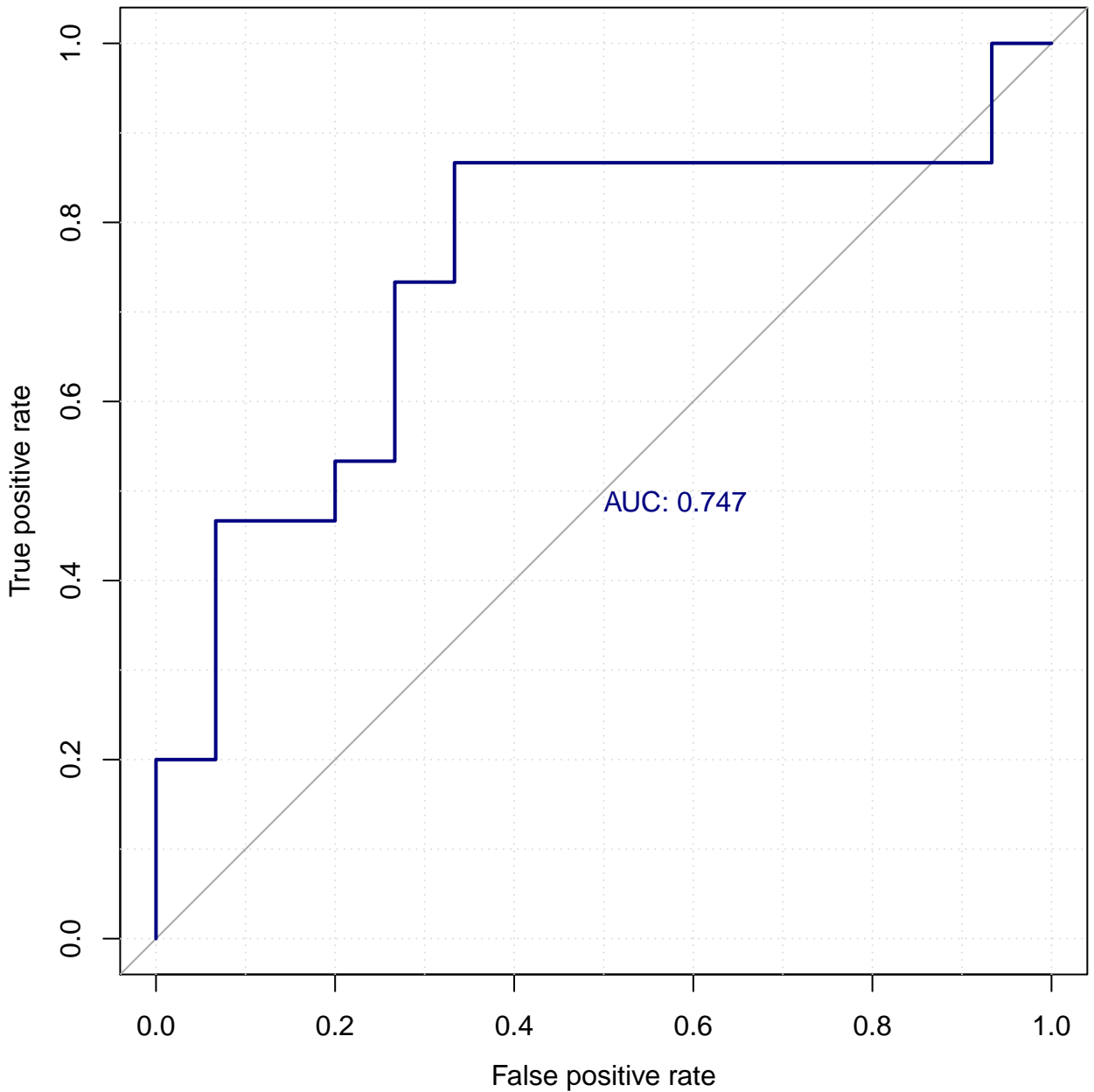

Supplement: Supplementary file 1 [file DataSheet7.PDF]

PDR.vs.T2DM

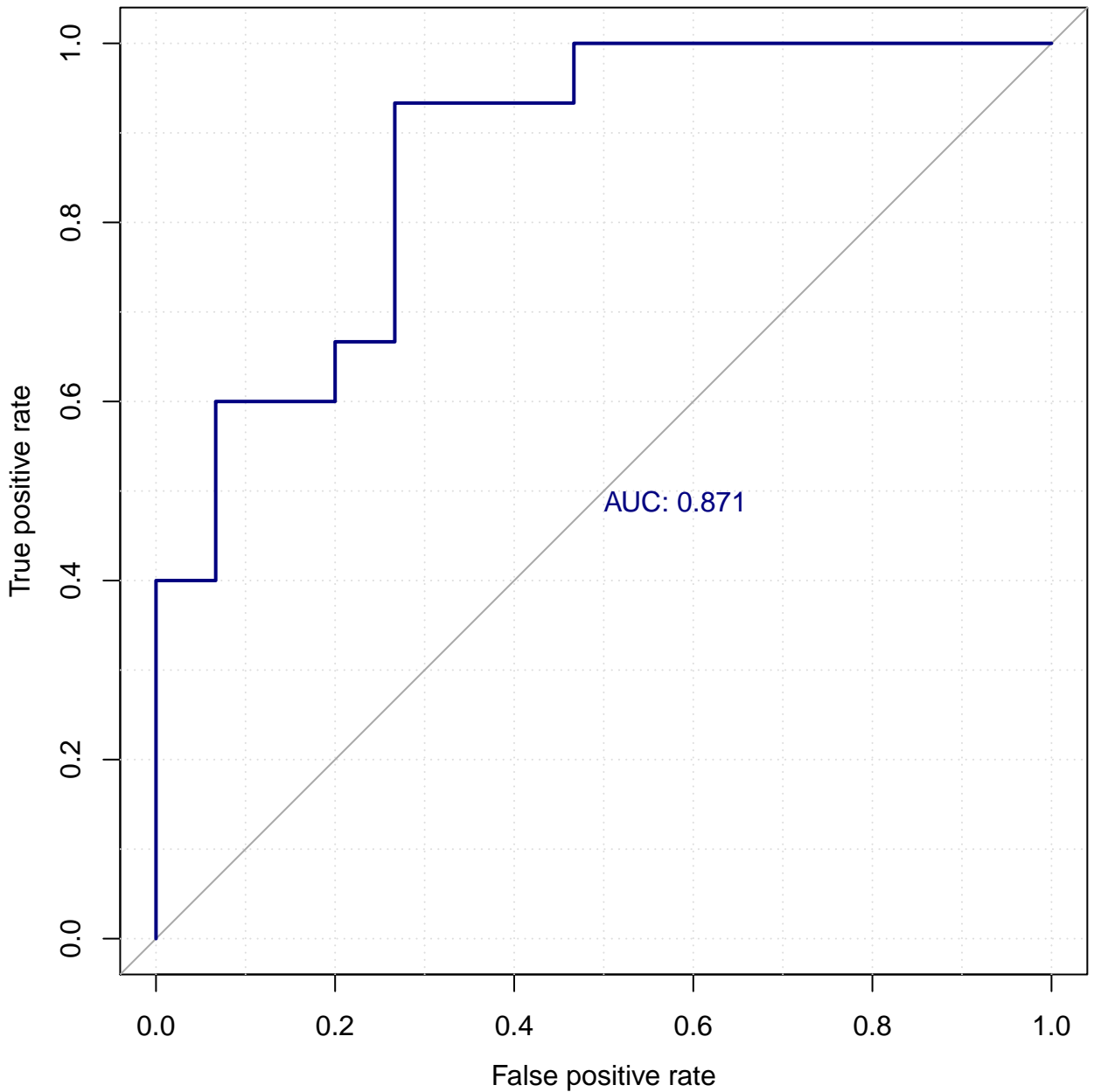

Supplement: Supplementary file 2 [file DataSheet13.PDF]

**PDR.vs.NPDR**

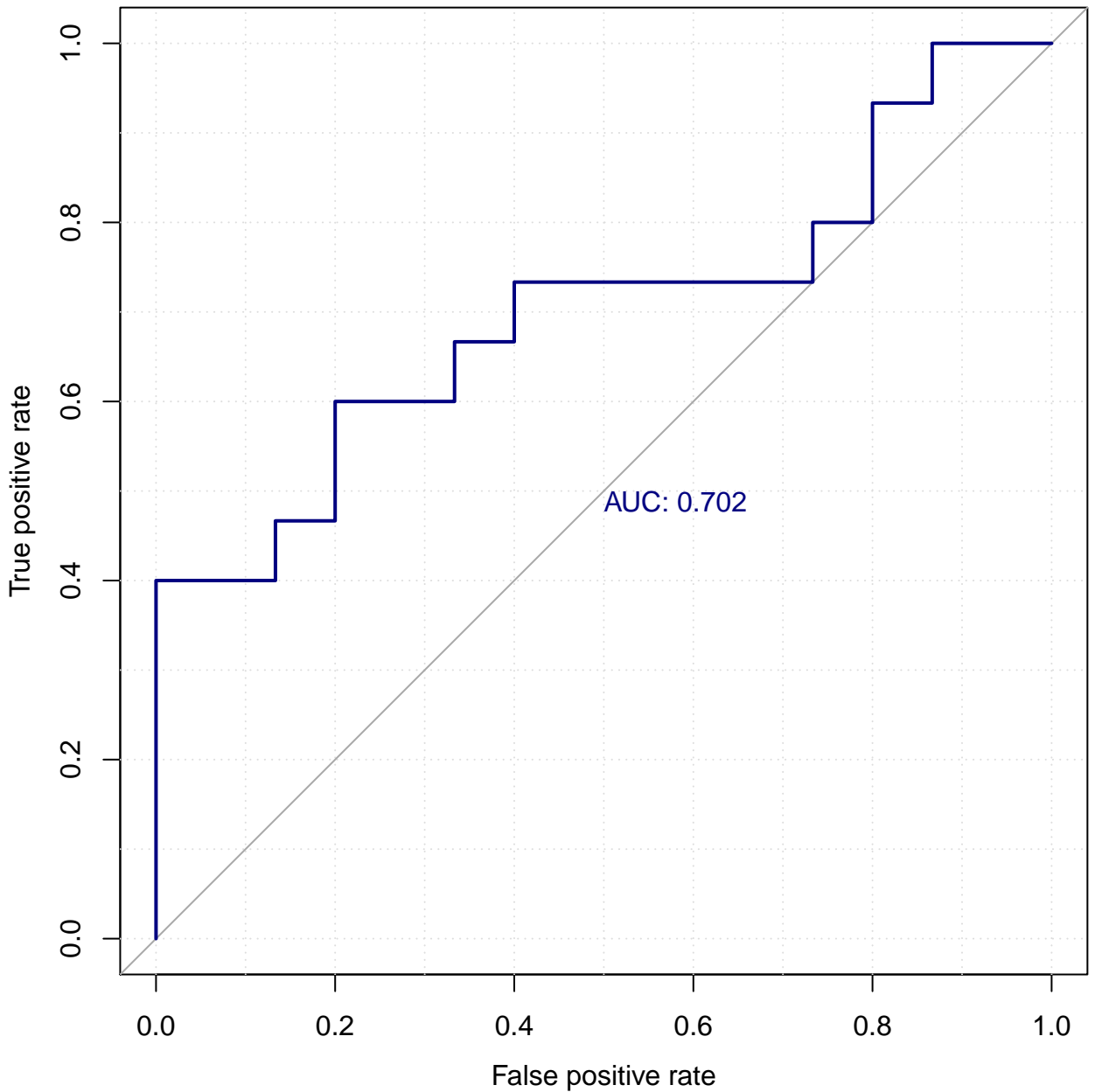

Supplement: Supplementary file 3 [file DataSheet2.PDF]

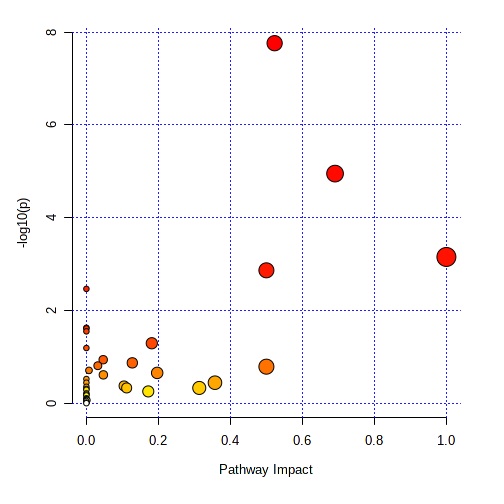

Supplement: Supplementary file 4 [file Image3.JPEG]

**PDR.vs.NPDR**

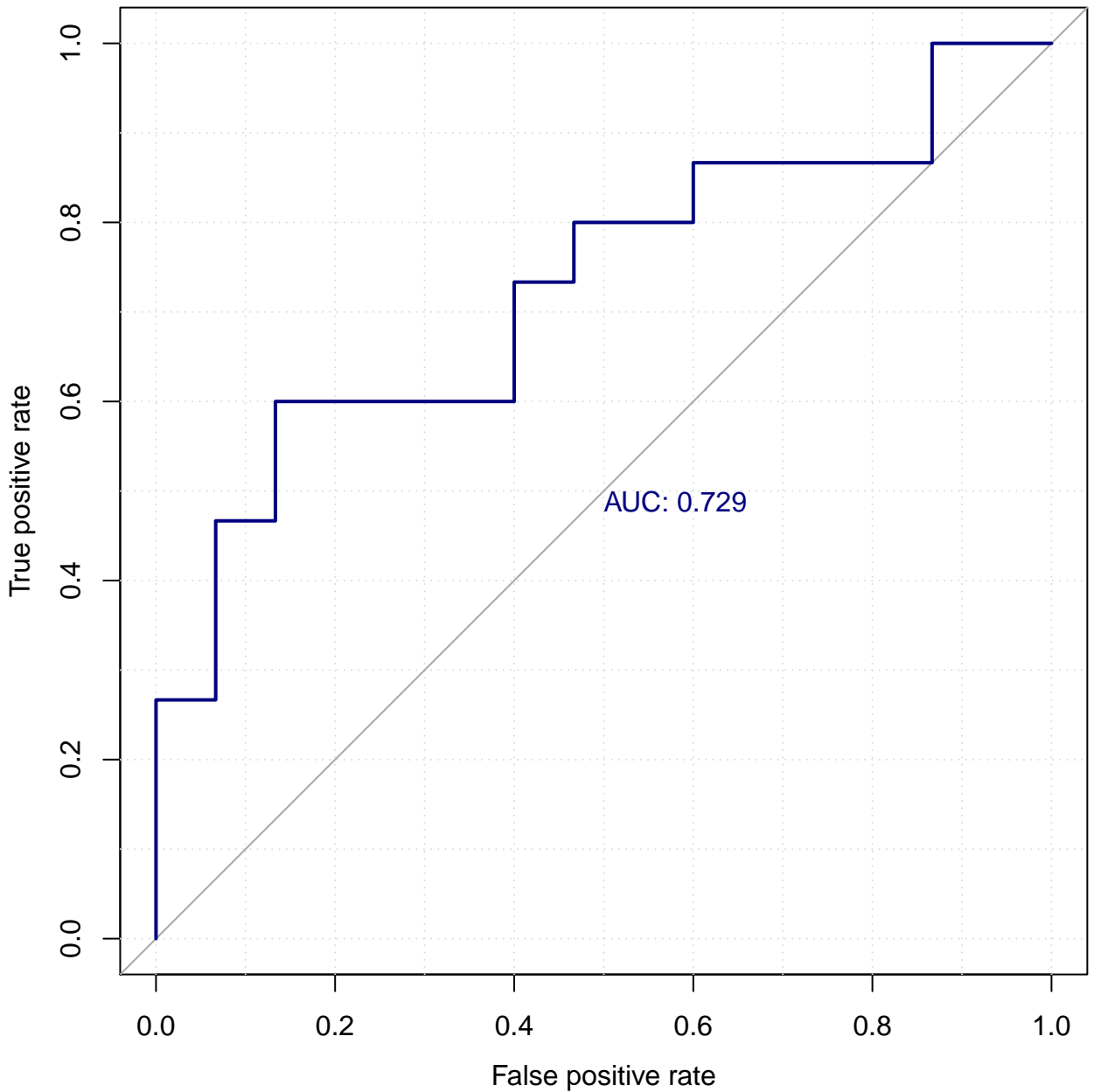

Supplement: Supplementary file 5 [file DataSheet4.PDF]

**PDR.vs.NPDR**

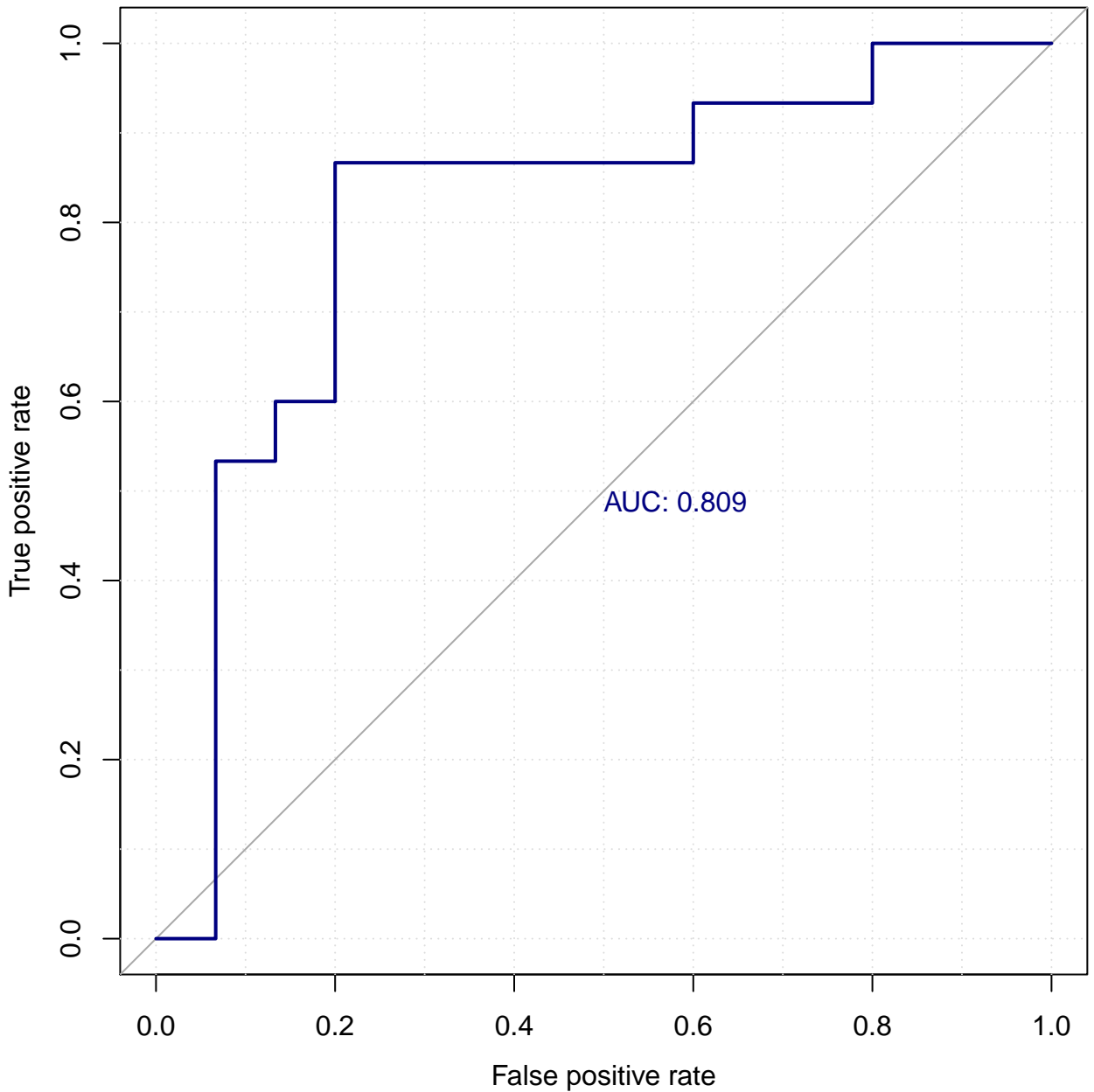

Supplement: Supplementary file 6 [file DataSheet6.PDF]

**PDR.vs.T2DM**

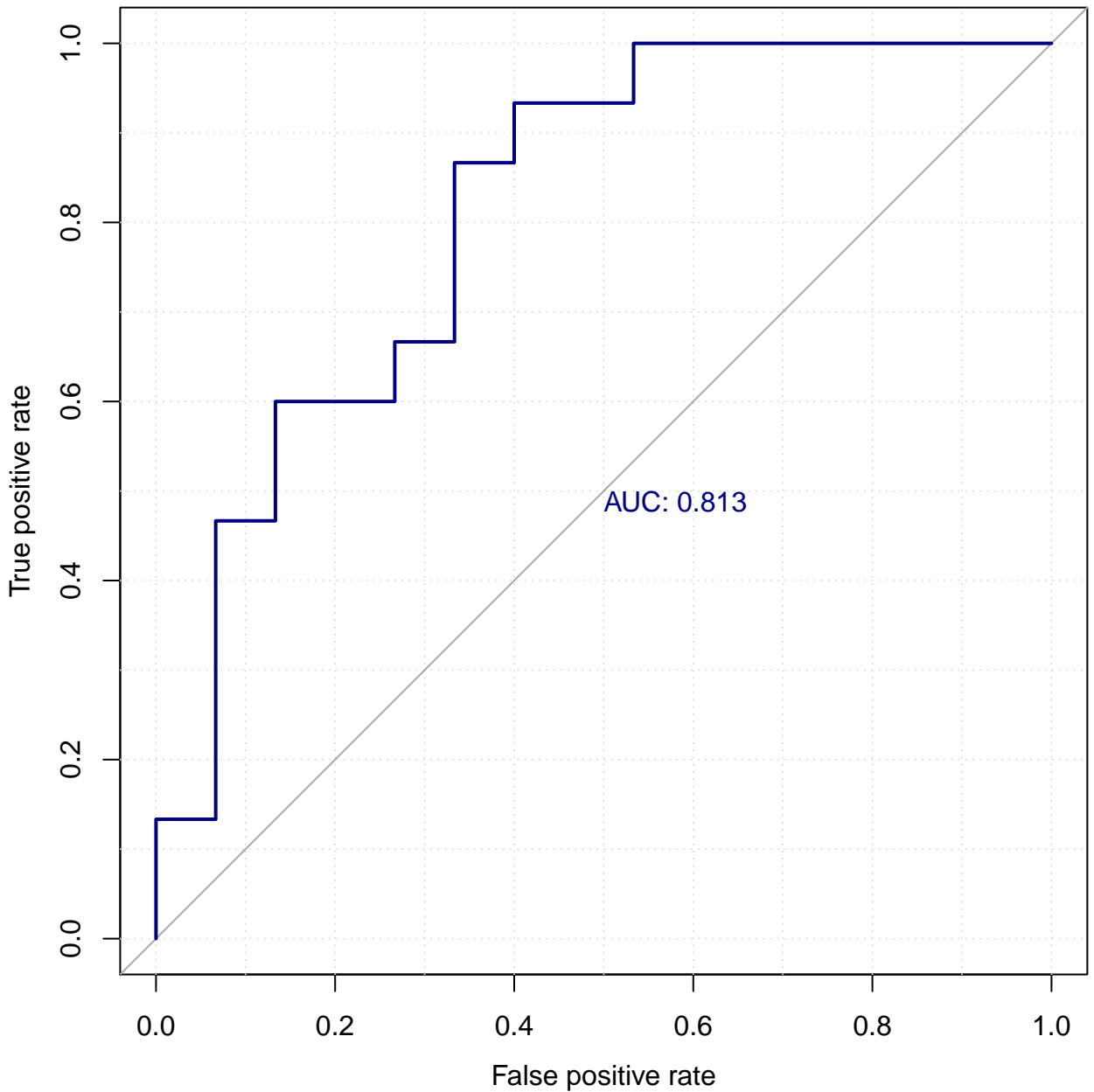

Supplement: Supplementary file 7 [file DataSheet14.PDF]

PDR.vs.T2DM

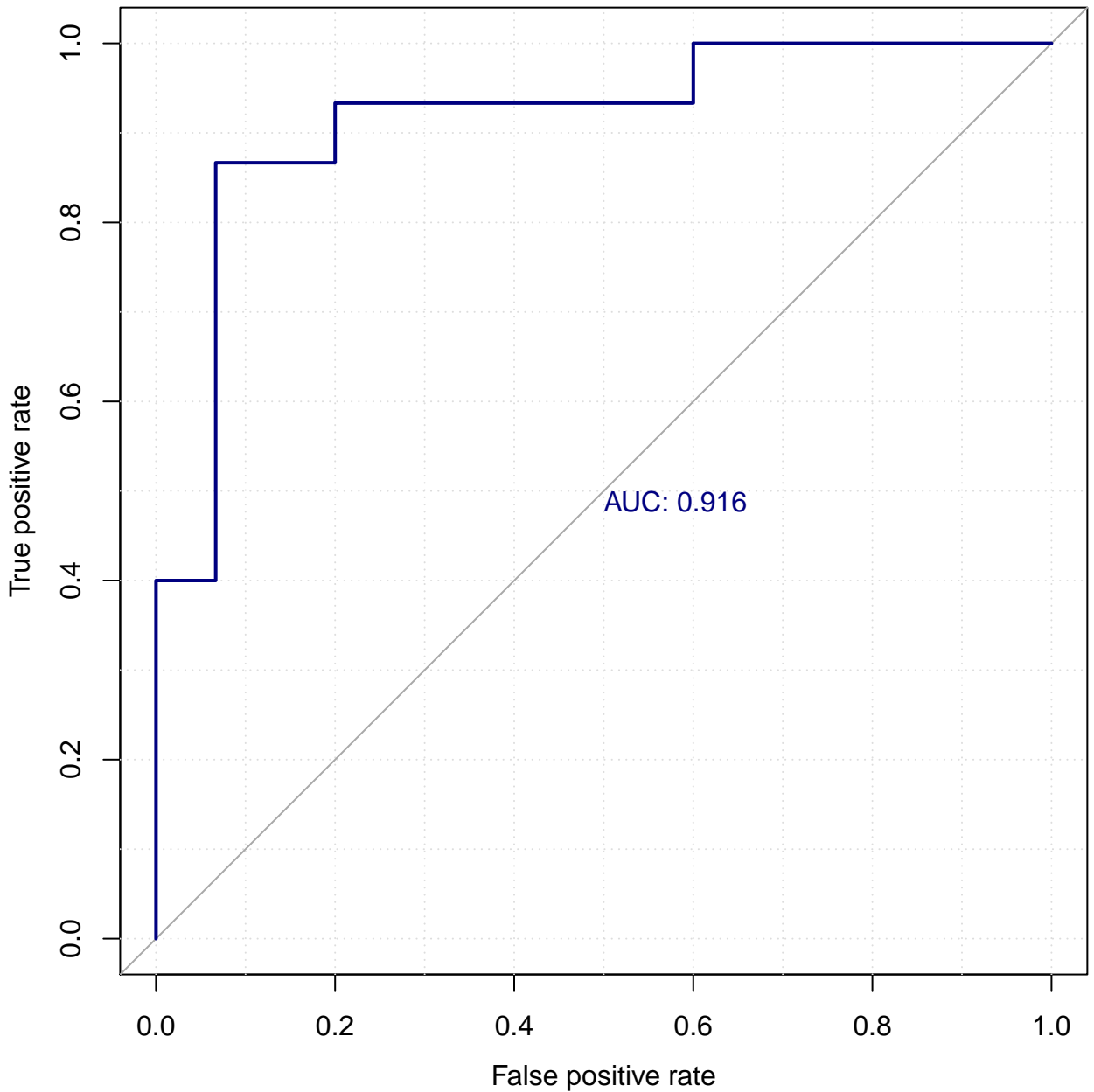

Supplement: Supplementary file 8 [file DataSheet9.PDF]

PDR.vs.T2DM

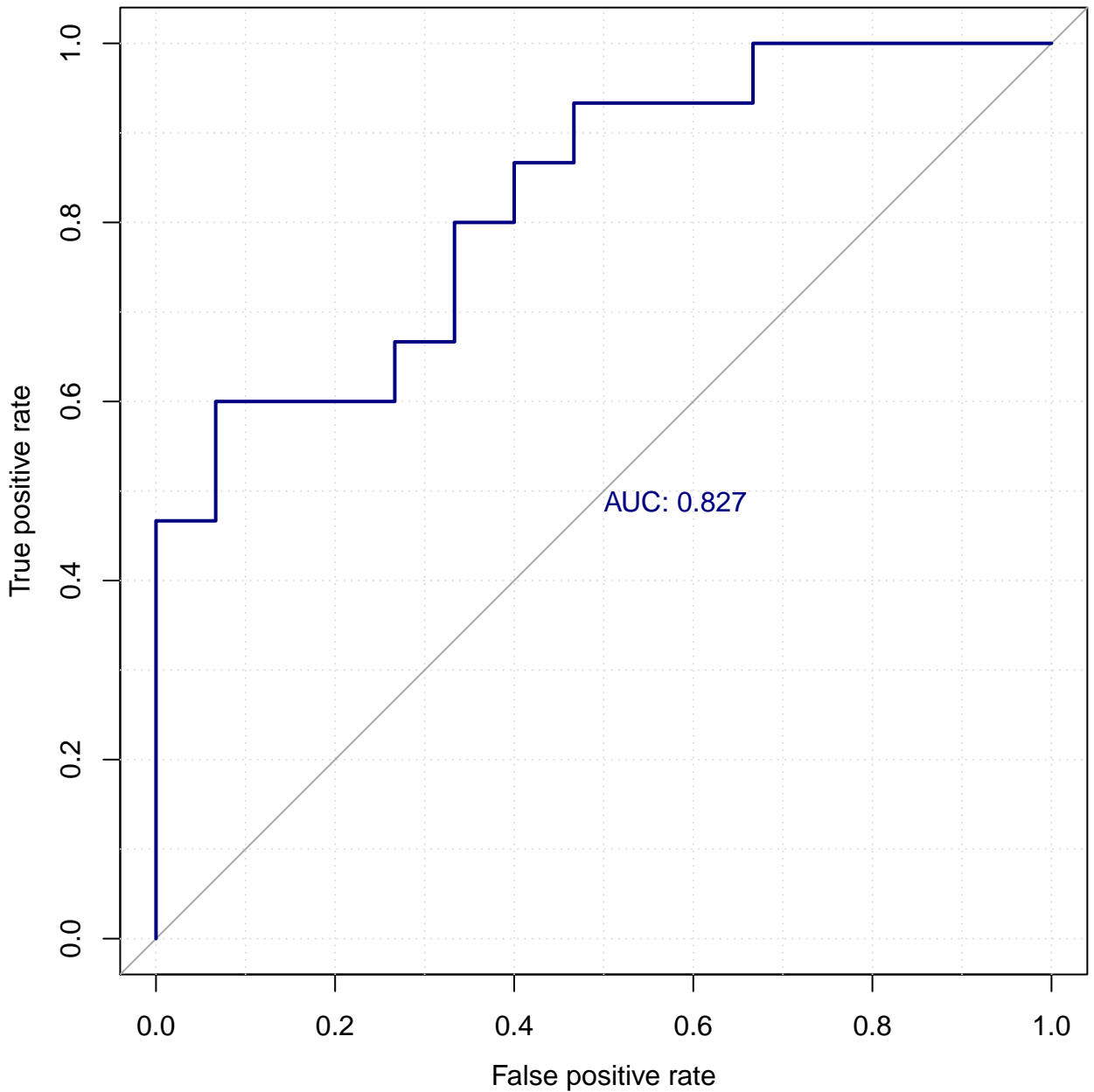

Supplement: Supplementary file 10 [file DataSheet11.PDF]

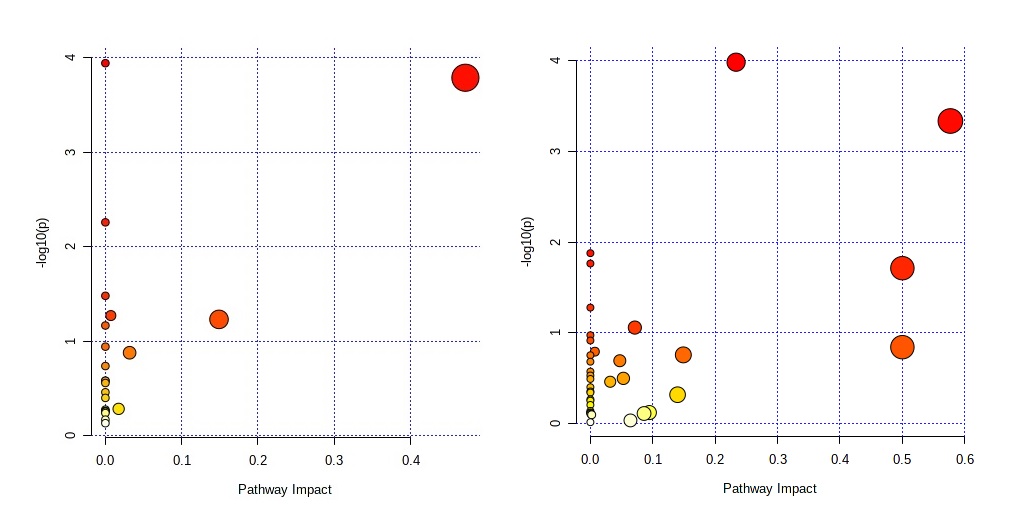

Supplement: Supplementary file 11 [file Image1.JPEG]

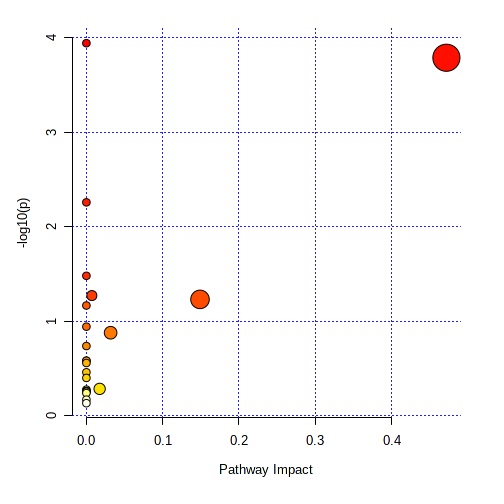

Supplement: Supplementary file 12 [file Image4.JPEG]

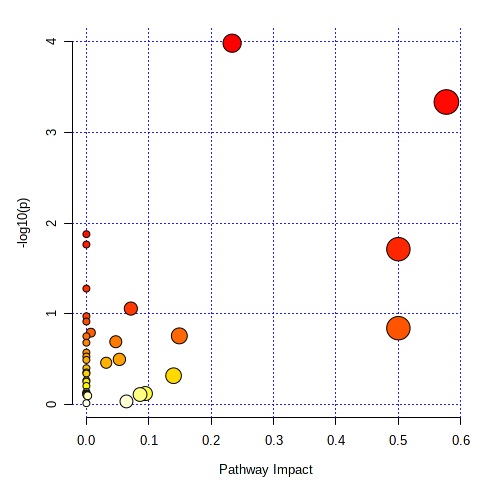

Supplement: Supplementary file 13 [file Image2.JPEG]

**PDR.vs.NPDR**

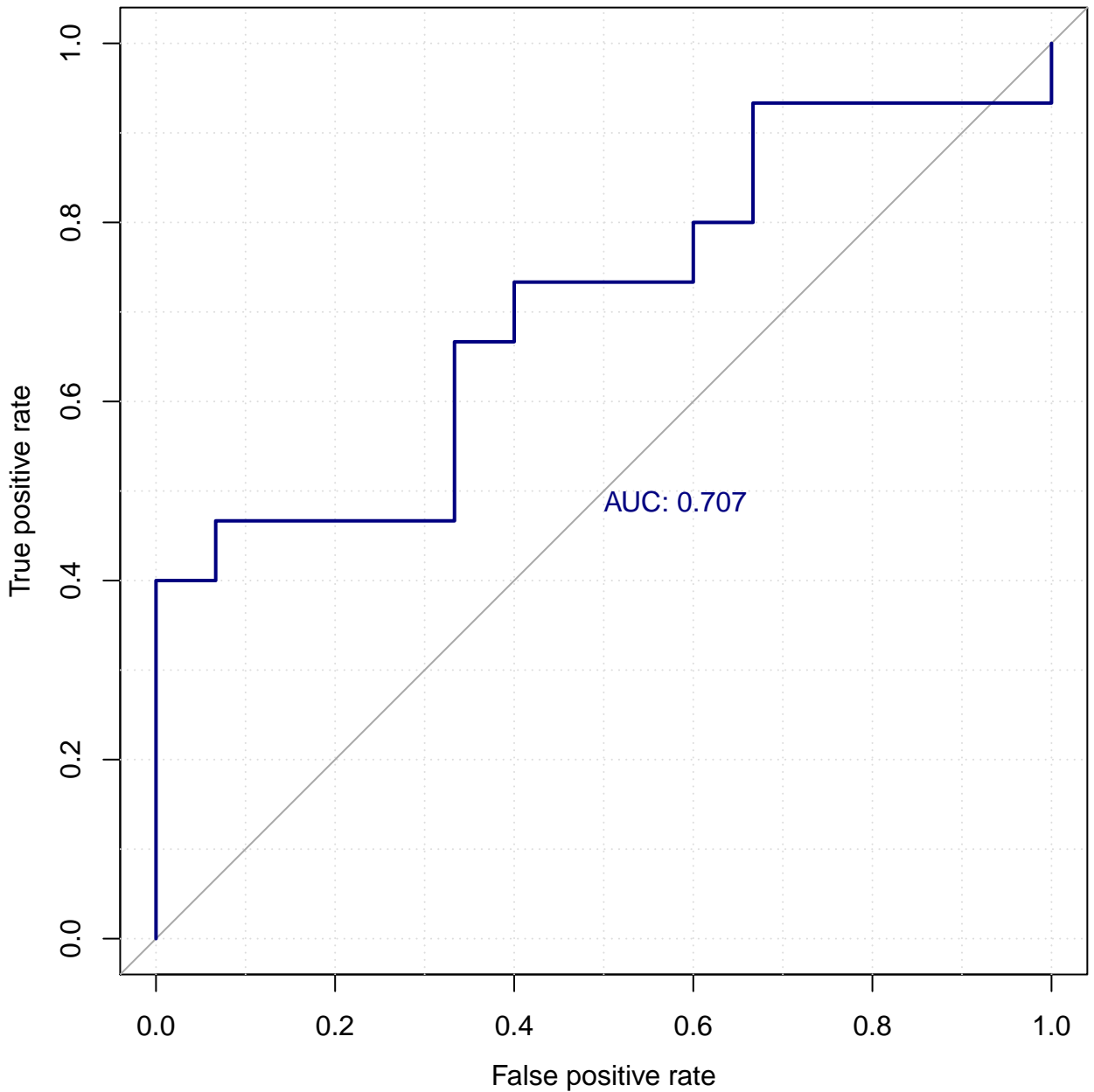

Supplement: Supplementary file 14 [file DataSheet3.PDF]

**PDR.vs.NPDR**

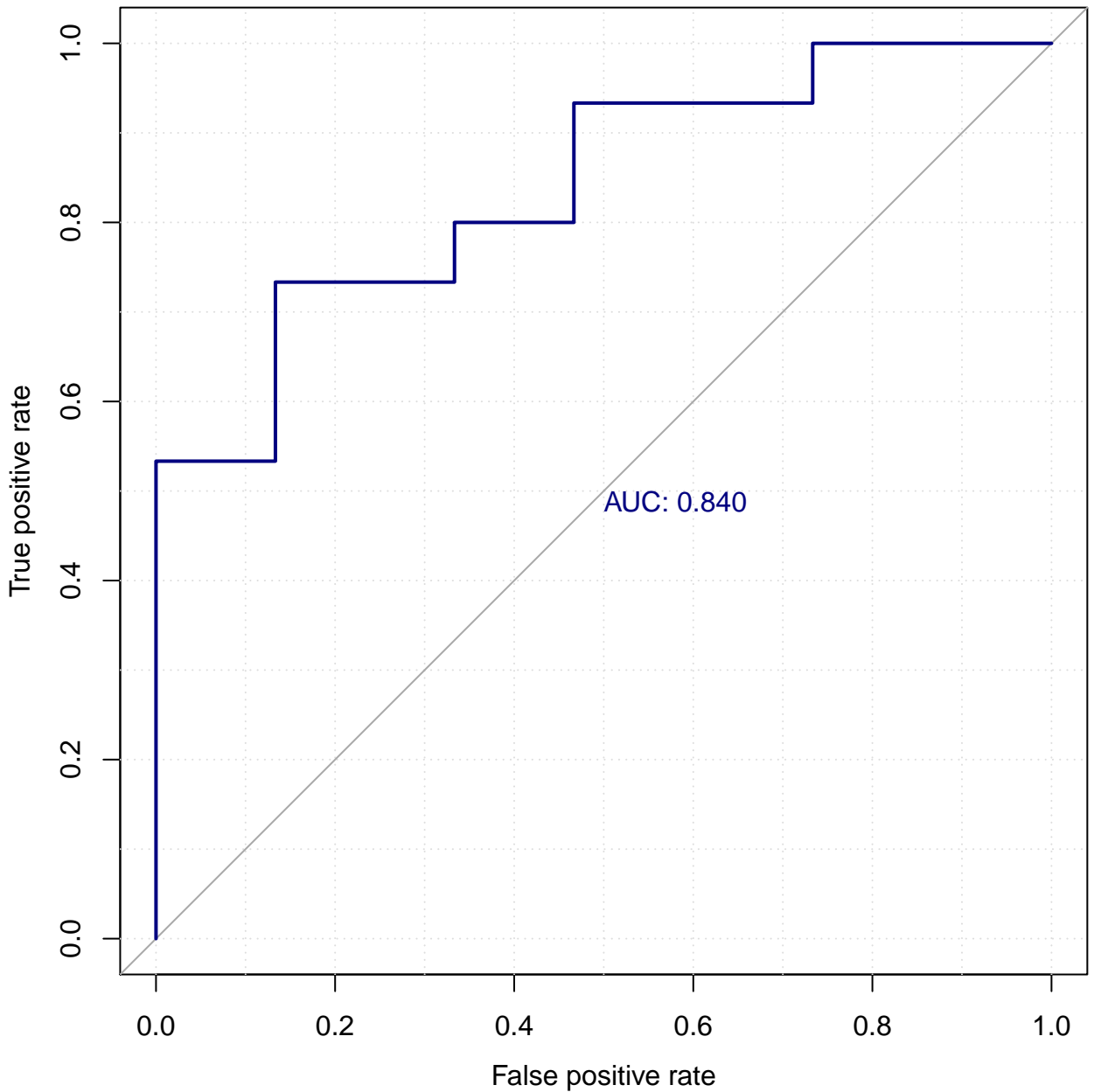

Supplement: Supplementary file 15 [file DataSheet1.PDF]

**PDR.vs.NPDR**

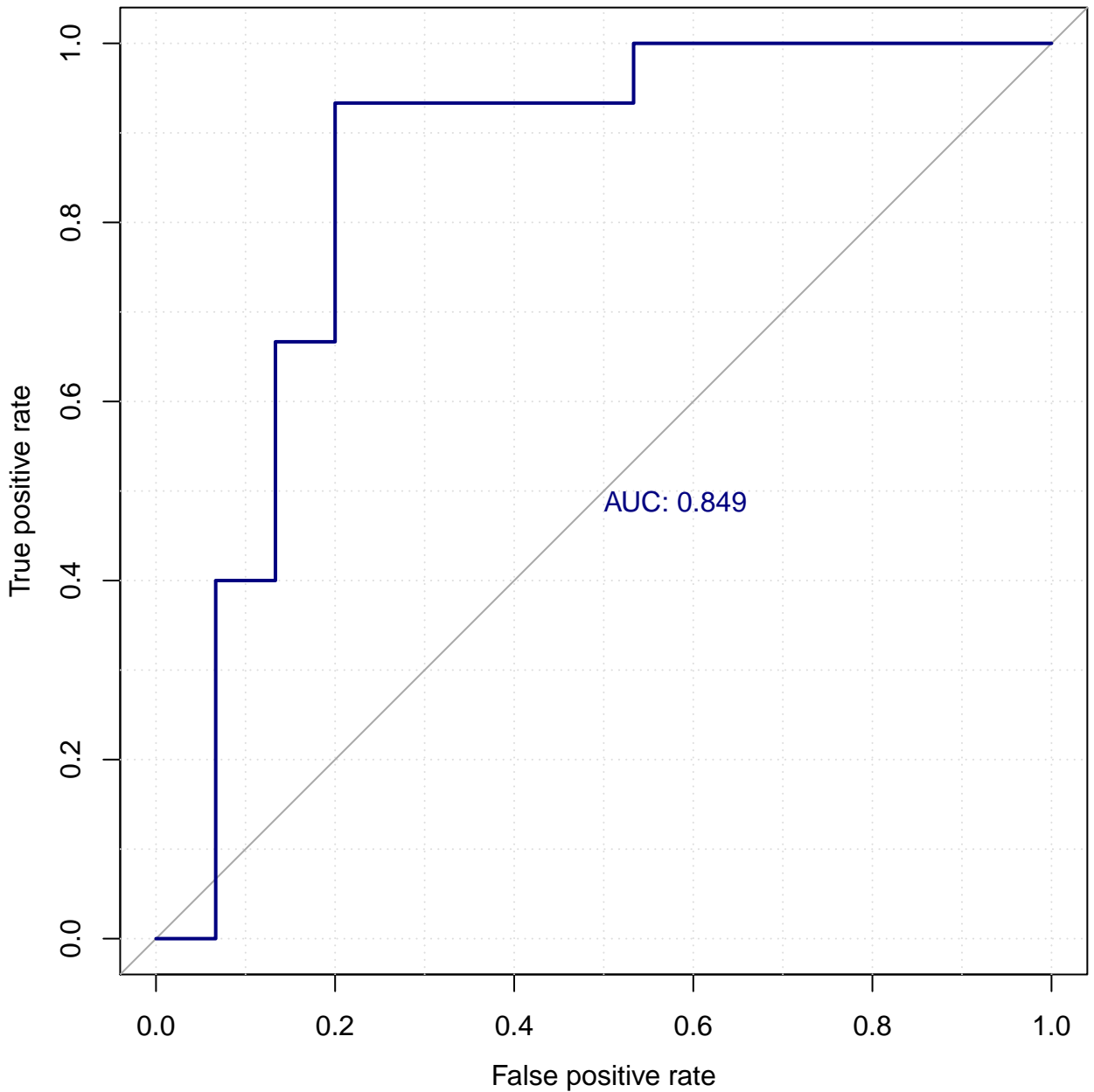

Supplement: Supplementary file 16 [file DataSheet5.PDF]

PDR.vs.T2DM

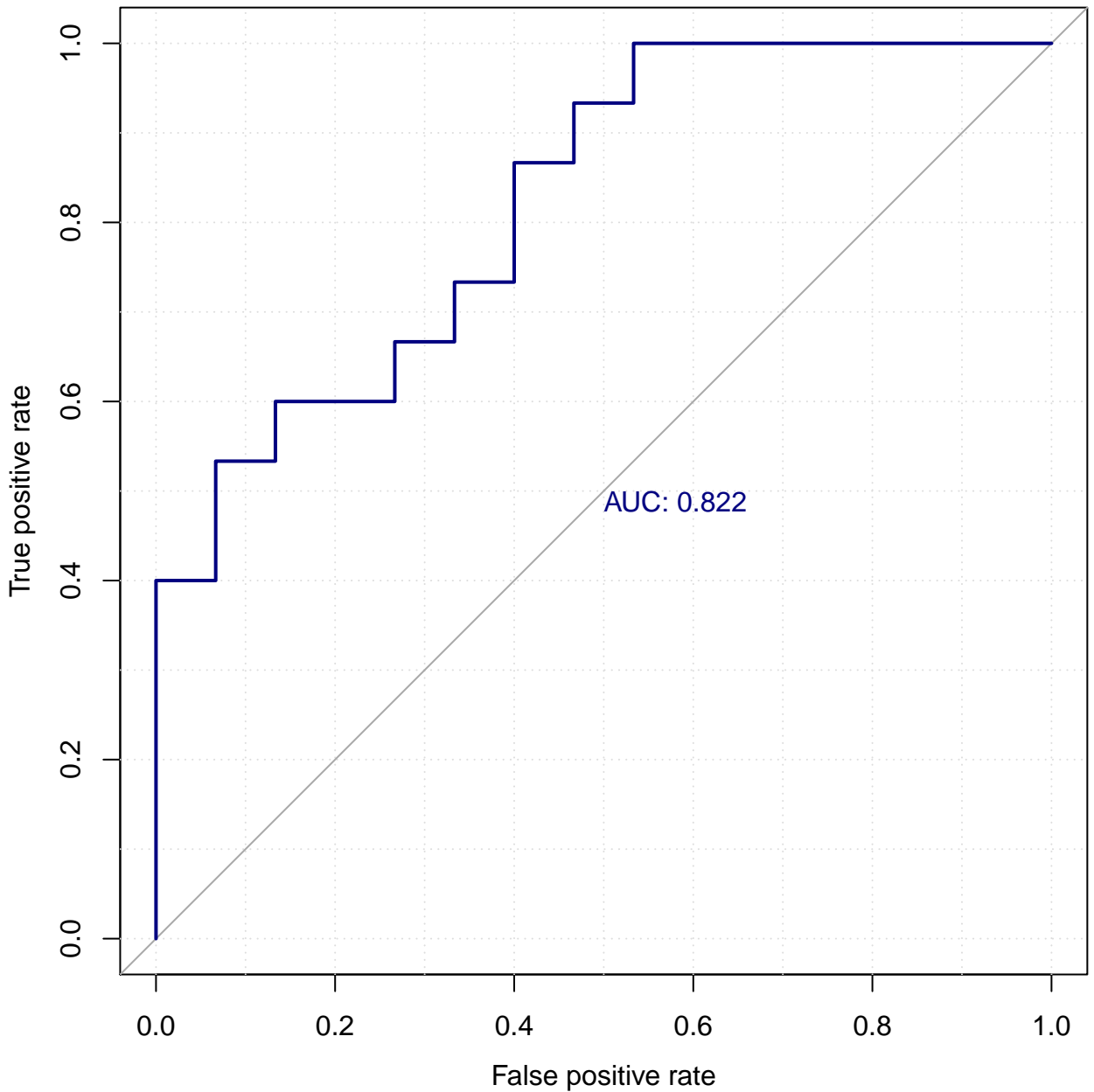

Supplement: Supplementary file 17 [file DataSheet12.PDF]

**PDR.vs.T2DM**

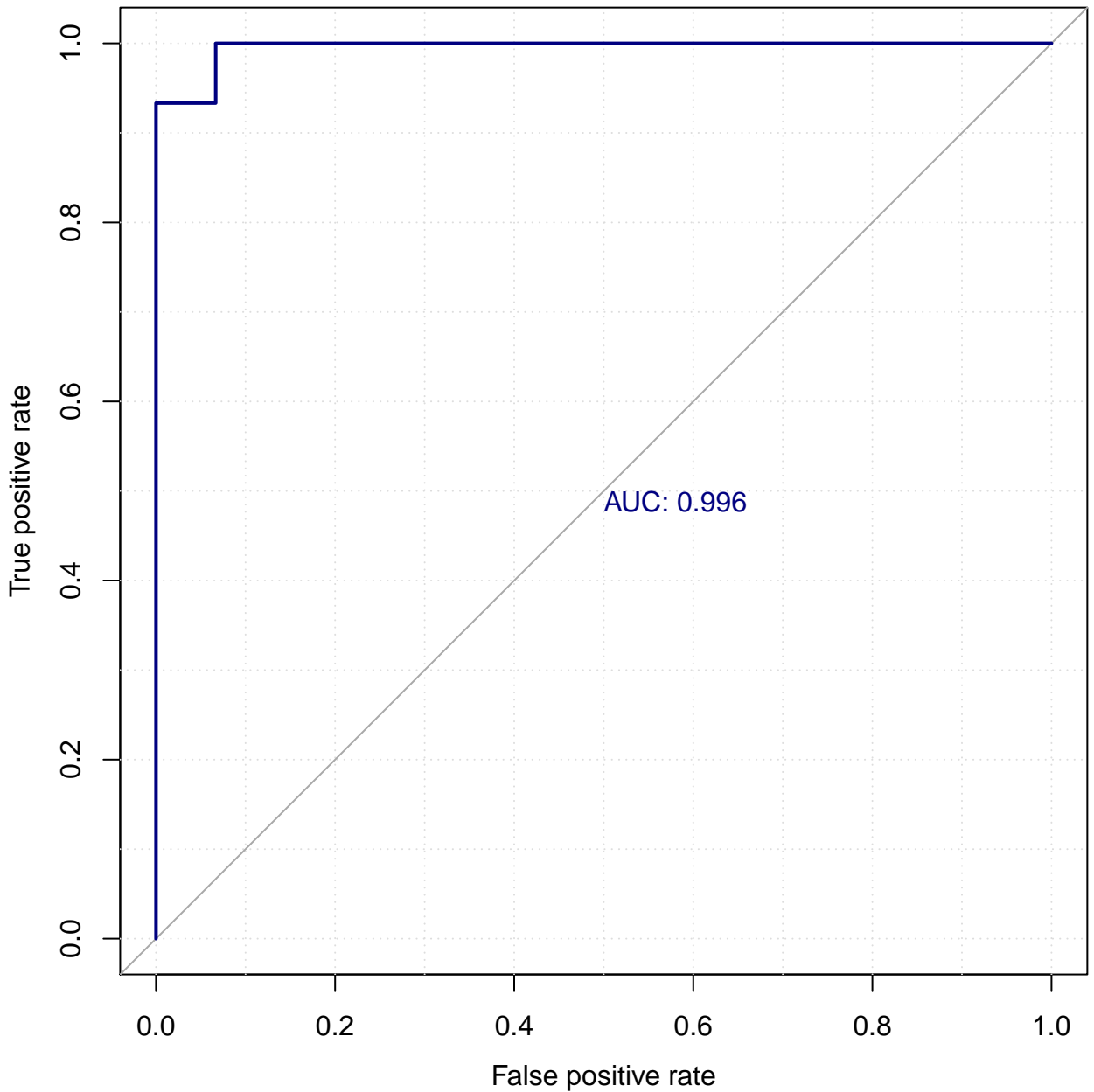

Supplement: Supplementary file 18 [file DataSheet8.PDF]

**PDR.vs.T2DM**

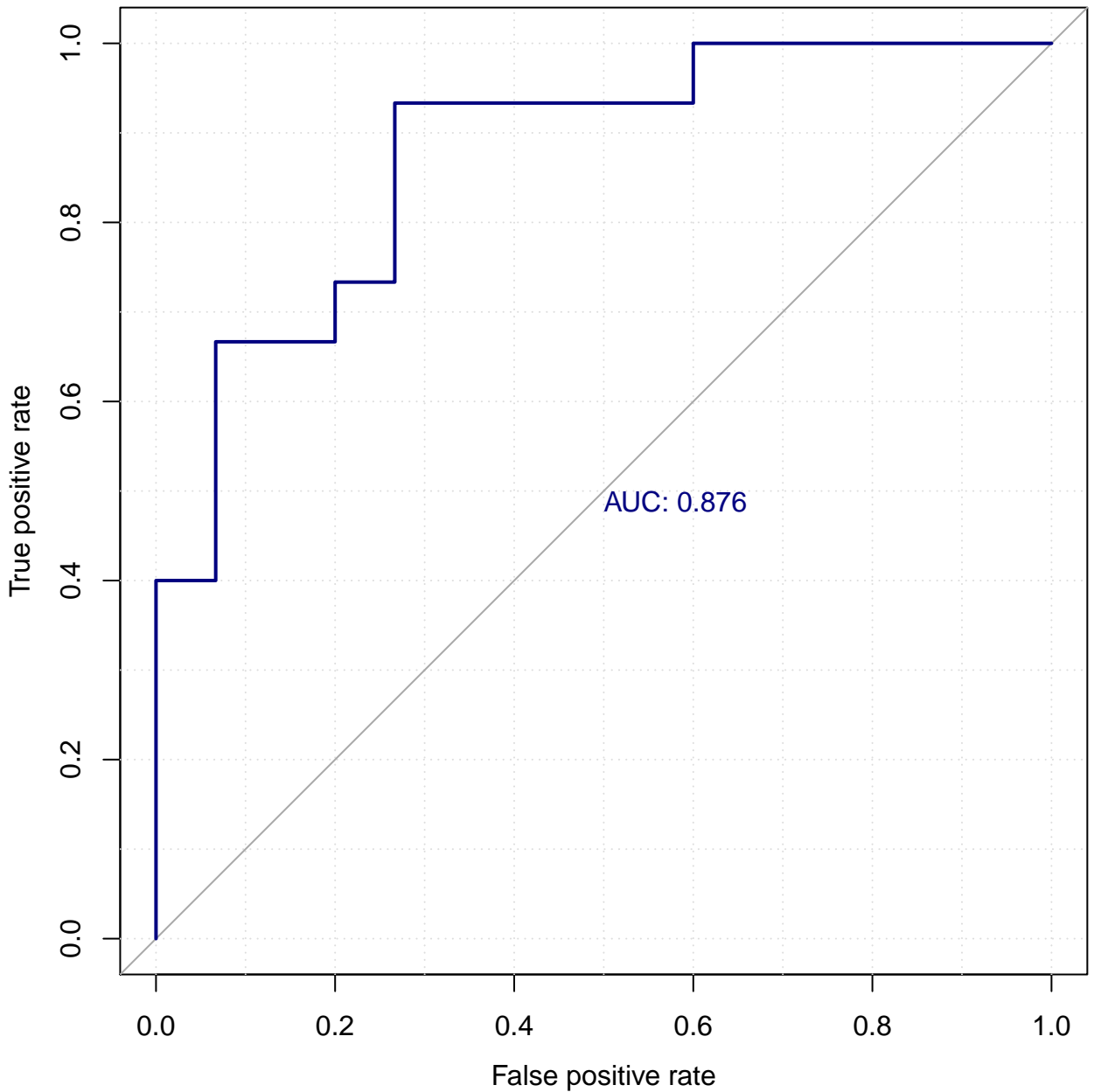

Supplement: Supplementary file 19 [file DataSheet10.PDF]
