# Supplementary material for: Serum Untargeted Metabolomics Reveal Potential Biomarkers of Progression of Diabetic Retinopathy in Asians
Source: Front Mol Biosci. 2022 Jun 9;9:871291. doi: 10.3389/fmolb.2022.871291 (PMC9224596; doi:10.3389/fmolb.2022.871291)
Supplement: Supplementary file 9 [file Table1.DOCX]

Supplementary Fig 1. Pathways Altered in NPDR Patients Compared With T2DM Patients. KEGG enrichment pathway analysis showed enrichment of 22 metabolic pathways between NPDR and T2DM.

Supplementary Fig 2. Pathways Altered in PDR Patients Compared With T2DM Patients. KEGG enrichment pathway analysis showed enrichment of 39 metabolic pathways between PDR and T2DM.

Supplementary Fig 3. Serum levels of (A) aspartate (P=0.001, AUC=0.996, VIP=1.52), (B) glutamate (P=1.70E-05, AUC=0.916, VIP=1.49), (C) glutamine (P=6.43E-05, AUC=0.876, VIP=1.25), (D) ornithine (P=9.50E-04, AUC=0.827, VIP=1.20), (E) 2-oxoglutarate (P=0.007, AUC=0.822, VIP=1.31), (F) N-acetyl-L-glutamate (P=0.002, AUC=0.871, VIP=1.52), (G) N-acetyl-L-aspartate (P=0.004, AUC=0.813, VIP=1.37), (H) citrate (P=0.011, AUC=0.813, VIP=1.26), (I) phosphatidylcholine (P=0.001, AUC=0.871, VIP=1.56), (J) 13-hydroperoxyoctadeca-9,11-dienoic acid (P=0.001, AUC=0.796, VIP=1.35), (K) methionine (P=0.001, AUC=0.840, VIP=1.08), (L) lysine (P=0.001, AUC=0.831, VIP=1.00), (M) threonine (P=0.002, AUC=0.836, VIP=1.20), (N) phenylalanine (P=0.010, AUC=0.751, VIP=1.06), (O) N-(L-arginino) succinate (P=0.026, AUC=0.796, VIP=1.51), (P) succinate (P=0.030, AUC=0.711, VIP=1.25) and (Q) N(pi)-methyl-L-histidine (P=0.031, AUC=0.716, VIP=1.45) were elevated in PDR patients. Serum levels of (R) linoleate (P=5.31E-04, AUC=0.871, VIP=1.01) significantly lower in PDR subjects. AUC, area under the curve.

Supplementary Fig 4. Pathways Altered in PDR Patients Compared With NPDR Patients. KEGG enrichment pathway analysis showed enrichment of 23 metabolic pathways between PDR and T2DM.

Supplementary Fig 5. Serum levels of (A) aspartate (P=0.004, AUC=0.853, VIP=1.78), (B) glutamine (P=6.73E-04, AUC=0.840, VIP=1.53), (C) N-Acetyl-L-glutamate (P=0.028, AUC=0.702, VIP=1.35), (D) N-Acetyl-L-aspartate (P=0.021, AUC=0.707, VIP=1.36), and (E) pantothenate (P=0.025, AUC=0.729, VIP=1.52) were elevated in PDR patients. Serum levels of (F) dihomo-gamma-linolenate (P=5.65E-04, AUC=0.849, VIP=2.36), (G) docosahexaenoic acid (P=0.005, AUC=0.809, VIP=1.28) and (H) icosapentaenoic (P=0.012, AUC=0.747, VIP=1.18) in PDR patients were significantly decreased. AUC, area under the curve.

Supplementary Fig 6. Pathways Altered in Both DR (including NPDR and PDR) versus T2DM and DR versus non-DR. KEGG enrichment pathway analysis showed enrichment of 35 and 37 metabolic pathways respectively between DR (including NPDR and PDR) versus T2DM and DR versus non-DR.
